# DNA Barcode Authentication and Library Development for the Wood of Six Commercial *Pterocarpus* Species: the Critical Role of Xylarium Specimens

**DOI:** 10.1038/s41598-018-20381-6

**Published:** 2018-01-31

**Authors:** Lichao Jiao, Min Yu, Alex C. Wiedenhoeft, Tuo He, Jianing Li, Bo Liu, Xiaomei Jiang, Yafang Yin

**Affiliations:** 1 Department of Wood Anatomy and Utilization, Chinese Research Institute of Wood Industry, Chinese Academy of Forestry, Beijing, 100091 China; 2 Wood Collections (WOODPEDIA), Chinese Academy of Forestry, Beijing, 100091 China; 3 Center for Wood Anatomy Research, USDA Forest Service, Forest Products Laboratory, Madison, WI 53726 USA; 4 Department of Botany, University of Wisconsin, Madison, WI 53706 USA; 5 Department of Forestry and Natural Resources, Purdue University, West Lafayette, IN 47907 USA; 6 Ciências Biológicas (Botânica), Univesida de Estadual Paulista – Botucatu, Botucatu, São Paulo Brazil; 7 Rubber Research Institute, Chinese Academy of Tropical Agricultural Science, Hainan, 571737 China

## Abstract

DNA barcoding has been proposed as a useful tool for forensic wood identification and development of a reliable DNA reference library is an essential first step. Xylaria (wood collections) are potentially enormous data repositories if DNA information could be extracted from wood specimens. In this study, 31 xylarium wood specimens and 8 leaf specimens of six important commercial species of *Pterocarpus* were selected to investigate the reliability of DNA barcodes for authentication at the species level and to determine the feasibility of building wood DNA barcode reference libraries from xylarium specimens. Four DNA barcodes (ITS2, *matK*, *ndhF-rpl32* and *rbcL*) and their combination were tested to evaluate their discrimination ability for *Pterocarpus* species with both TaxonDNA and tree-based analytical methods. The results indicated that the combination barcode of *matK* + *ndhF-rpl32* + ITS2 yielded the best discrimination for the *Pterocarpus* species studied. The mini-barcode *ndhF-rpl32* (167–173 bps) performed well distinguishing *P. santalinus* from its wood anatomically inseparable species *P. tinctorius*. Results from this study verified not only the feasibility of building DNA barcode libraries using xylarium wood specimens, but the importance of using wood rather than leaves as the source tissue, when wood is the botanical material to be identified.

## Introduction

Increasing concern about and demand for biodiversity conservation world-wide and substantial declines in biological diversity at various spatial, temporal and biological scales^1^ are driving the need for species identification for forensics. For forest systems, illegal logging and the illegal timber trade are major problems domestically and internationally, threatening not just individual species, but entire ecosystems. Illegal logging is both a consumer- and producer-country driven phenomenon, and international efforts to respond to the problem consist of the enactment of laws to prohibit or limit the trade in illegally sourced timber. Broad international trade restrictions are imposed primarily through the Convention on International Trade in Endangered Species of Wild Fauna and Flora (CITES), which lists species in three appendices according to the degree of protection required^2^.

In recent years, several consumer countries and regions have also taken action to reduce the trade in forest products derived from illegally logged sources^3^. The United States amended the Lacey Act in 2008, which makes it unlawful to import into the United States any plant (or plant product) that was illegally harvested. In Australia, the Illegal Logging Prohibition Act (2012) was enacted to restrict the trade of illegally logged timber. The European Union’s E.U. Timber Regulation (EUTR) came into effect in 2013, prohibiting illegally sourced timber and timber products in the EU market. These legislative actions and subsequent enforcement of these laws demonstrate the urgent global attention on forest species protection. Enforcement actions to date in the United States have largely focused on ebony and rosewood from Madagascar (Dept. of Justice 2012), Eurasian hardwoods (Dept. of Justice 2016), and tropical woods from Peru (Dept. of Justice 2017) indicating that wood forensic methods, including DNA barcoding reference libraries, for valuable woods from anywhere in the world could play a critical role in law enforcement for forest protection.

*Pterocarpus* Jacq., is a pantropical genus in the family Leguminosae, containing approx. 70 species^4^. The timber of *Pterocarpus* is globally valued for its beauty, wood quality, medicinal properties and even valuable bioactive compounds. This high value and increase in demand for the timber has led to illegal and excessive logging resulting in threat to wild *Pterocarpus* populations. In 1995, CITES listed *P. santalinus* under Appendix II to regulate trade in logs, wood chips and unprocessed broken material^5^. *P. erinaceus* was added to Appendix II at the 17th Meeting of the CITES in 2017. Concurrently, the International Union for Conservation of Nature (IUCN) also listed *P. santalinus* and *P. zenkeri* as endangered, *P. indicus* and *P. marsupium* as vulnerable, and *P. angolensis* as near threatened^6^. In China, the species *P. indicus* was listed in the second-class category of the National List of Local Protected Flora issued by the Chinese Government in 1999^7^. Among the *Pterocarpus* species, *P. santalinus*, endemic to the Southern parts of Eastern Ghats of India especially in Andhra Pradesh, is known for its characteristic color, texture, quality and the medical value of its timber, which makes it of particular economic importance, especially in China. In recent years, the wood from *P. tinctorius* (non-CITES) mostly distributed in Central and Southern Africa, appeared on the international lumber market as a substitute for *P. santalinus* (CITES App. II). Its macroscopic wood properties, e.g. color, grain, density, and its anatomical structure are very similar to that of *P. santalinus*. Due to the great difference in economic value, *P. tinctorius* has often been treated as an adulterant of *P. santalinus* in the timber market. Thus, developing accurate species-level identification for *Pterocarpus* wood is significant for natural resource protection and global trade monitoring.

Traditional wood identification relies on diagnostic anatomical features, either macroscopic or microscopic but rarely can provide a precise discrimination of wood at the species level, which limits the enforcement of CITES regulations and related laws. Moreover, traditional wood identification requires expert taxonomic and anatomical knowledge that takes years to gain. To overcome such limitations, recent advances in molecular diagnostic tools for plants have the capacity to improve upon traditional methods of species identification.

For the last decade, DNA barcoding has been the subject of extensive research and application as an accurate and convenient tool for species identification^8–12^. DNA barcoding is a genetic approach based on a short DNA sequence from a standard part of a genome – in animals this is typically a region of the cytochrome c oxidase subunit 1 (CO1) mitochondrial region. In plants, mitochondrial mutation rates are too slow for species-level identification, so plastid and nuclear regions are typically chosen as barcodes^13,14^. The Consortium for the Barcode of Life (CBOL) proposed a combination of both the chloroplast DNA (cpDNA) ribulose-bisphosphate carboxylase (*rbcL*) gene and maturase K (*matK*) genes as the core DNA barcodes for plants. Chen *et al*.^15^ proposed that the ITS2 region could be potentially used as a standard DNA barcode, especially for identifying medicinal plants and their closely related species^15^. Additionally, the *ndhF-rpl32* intergenic spacer in the short single copy region of the chloroplast genome, which was noted as highly variable^16^ by Timme *et al*.^16^, has also been used for phylogenetic studies^17,18^. DNA barcodes are established tools for identifying herbal medicinal materials, in quality control, and in forensic science^10,19,20^. Additionally, a number of studies relying upon DNA barcoding have verified the utility and potential for wood species identification^11,12,21–23^.

**Figure 1:**
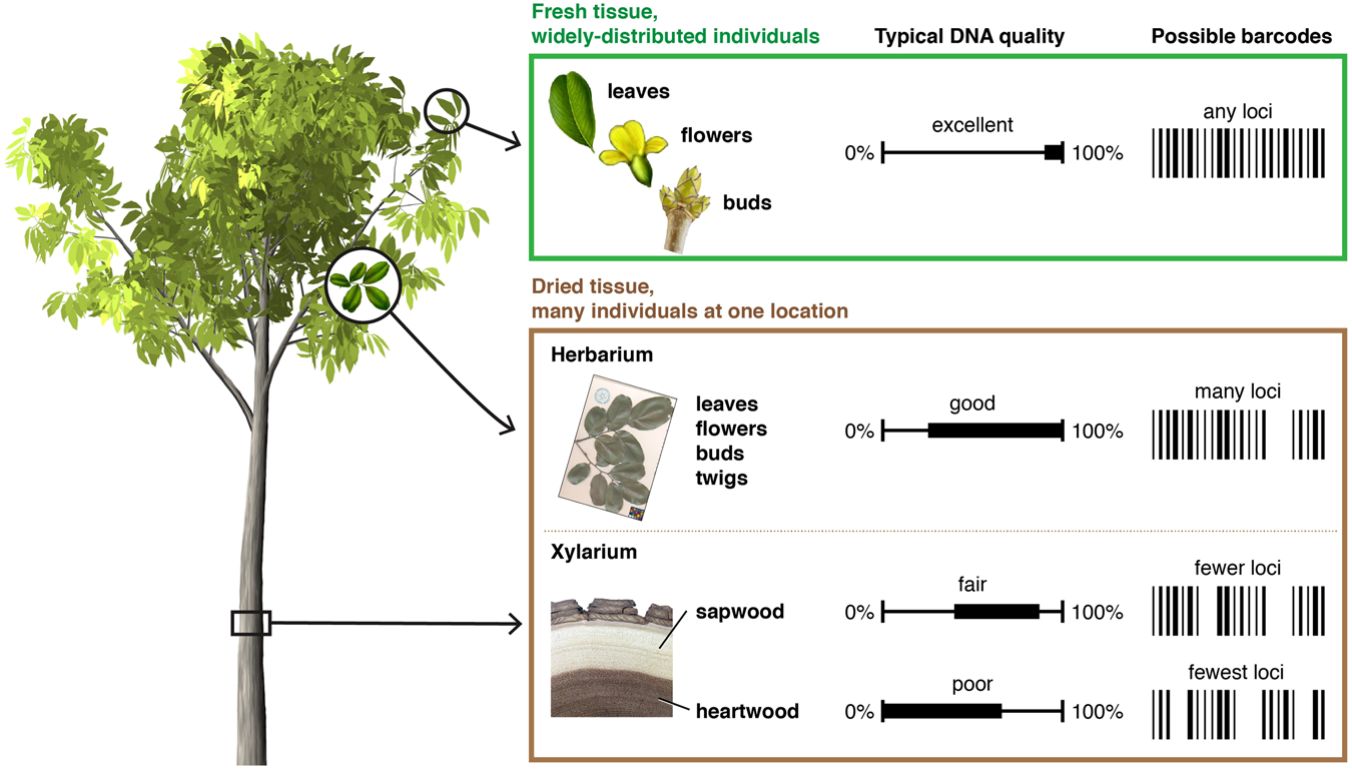
A schematic representation of the potential strengths and weaknesses of source tissue (fresh, herbarium, xylarium) for developing DNA barcoding reference libraries.

Despite the desirability of using DNA barcoding broadly in plant forensics, the lack of a reliable DNA barcoding reference library is the main barrier to its application for the next few years^24–28^. To generate such a reference library, access to correctly identified specimens of the species of interest is required. If these specimens are living individuals, extracting DNA of sufficient quality and quantity is routine, but for a widely-distributed taxon would involve significant expense and time to travel and sample across the taxon’s range. An alternative to this approach is to sample from botanical collections like herbaria^28^, where specimens of many taxa are gathered in one place, but from which high quality DNA may not be available. It is one step more complicated to develop a reference library for DNA barcoding of wood, because DNA extraction from wood is not necessarily as simple or direct as from other plant parts that can be collected and analyzed in the living state (Fig. 1). Because wood is a botanically poor source of DNA even prior to industrial processing, developing DNA reference libraries for wood discrimination is most sensibly done from scientifically collected wood specimens from xylaria – this ensures that extraction protocols, chosen barcodes, and developed methods are directly applicable to wood as a commercial product. There are approx. 180 xylaria containing on the order of 1.5 million wood specimens in the world^29^. Historically, xylaria played an important role in the development of wood science and priority forestry programs, as resources supporting timber trade, law enforcement, archaeology, and conservation and restoration of architectural wood heritage. Xylaria still serve these functions, but they also have potential to be enormous resources for DNA studies, providing abundant and reliable resource materials for establishing DNA barcoding reference databases, though few researchers to date have taken advantage of xylaria in this way^11^.

In this study, we selected xylarium wood specimens of six important commercial *Pterocarpus* species and evaluated four candidate DNA barcodes (ITS2, *matK*, *ndhF-rpl32*, and *rbcL*) for their efficacy at species-level separation. The specific objectives were to (i) test the discrimination ability of the barcodes using both TaxonDNA as well as a tree-based method, (ii) determine the efficacy of the mini-barcode *ndhF-rpl32* to separate *P. santalinus* from *P. tinctorious*, (iii) provide the essential data for establishing a reference library for *Pterocarpus* using the chosen barcodes individually and in combination, and (iv) verify the feasibility of building wood DNA barcode reference libraries using wood specimens from a xylarium.

## Results

### Wood anatomical separation of *P. santalinus* and *P. tinctorius*

**Figure 2:**
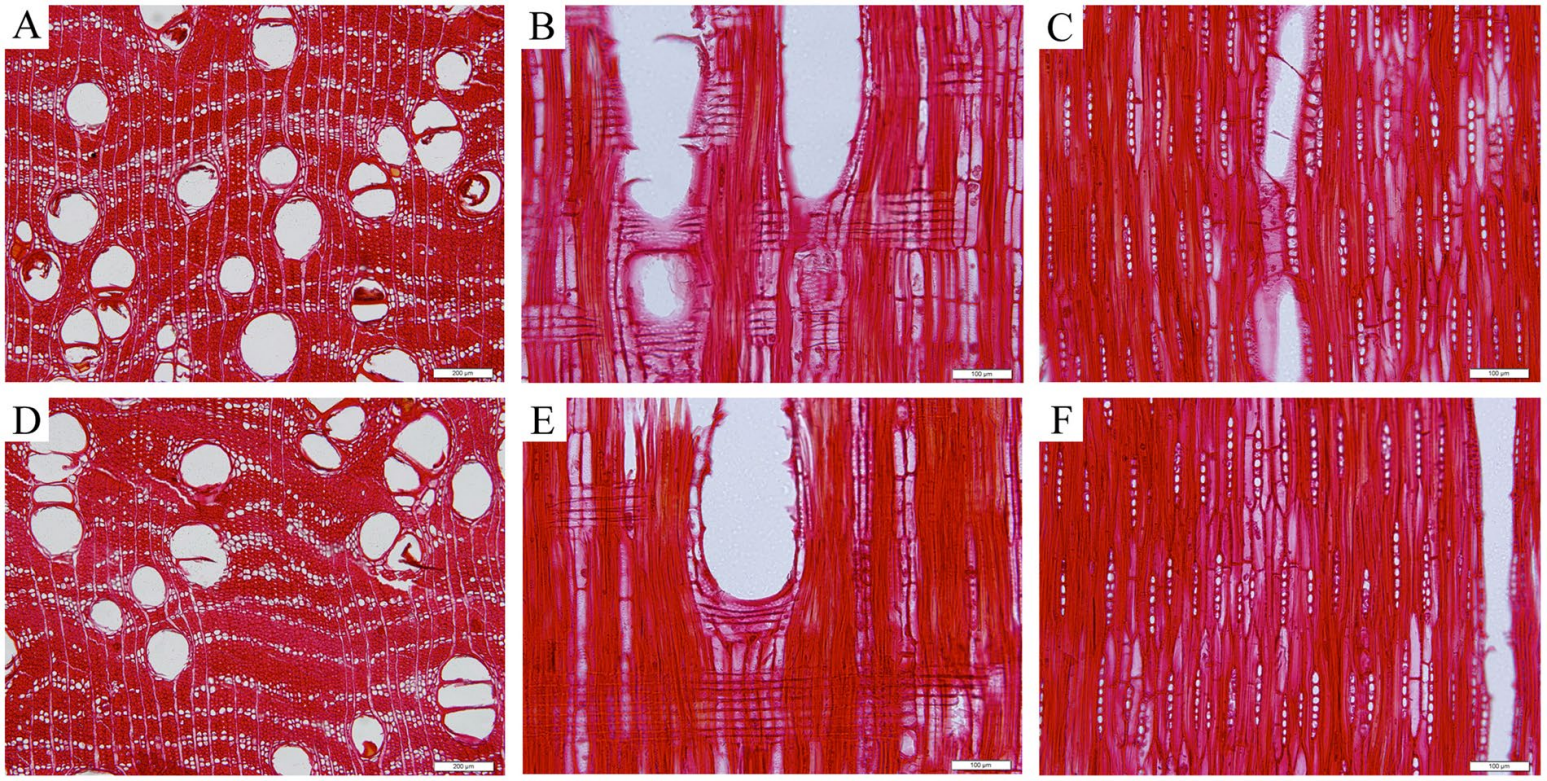
Wood anatomical features of *P. santalinus* and *P. tinctorius*. (**A**,**B** and **C**) Transverse, radial, and tangential sections of *P. santalinus* wood, respectively. (**D**,**E** and **F**) Transverse, radial, and tangential sections of *P. tinctorius* wood, respectively. Scale bars, 200 μm (**A** and **D**) and 100 μm (**B**,**C**,**E** and **F**).

The wood anatomical features of *P*. *santalinus* and *P. tinctorius* are almost identical (Fig. 2). Wood diffuse-porous; vessels exclusively solitary, occasionally with radial multiples of 2 to 3, and often filled with dark gums; intervessel pits alternate; vessel-ray pits similar to intervessel pits; perforation plates simple; axial parenchyma, aliform, confluent and narrow bands of 1–4 cells wide; prismatic crystals in chambered axial parenchyma cells; axial parenchyma cells storied; fibres thick-walled, storied; rays exclusively uniseriate, occasionally 2 cells wide, 2 to 10 cells high, homocellular, consisting of procumbent cells; All rays storied. In addition to these anatomical similarities, we report that ethanol extract color, heartwood surface fluorescence, heartwood water extract fluorescence, and heartwood ethanol extract fluorescence are all also indistinguishable. It is thus impossible to make a forensically valid separation of *P. santalinus* from *P. tinctorius* based on wood anatomical features.

### Barcode Recovery and Sequence Characteristics

The recovery success rate was the highest for *ndhF-rpl32* (90%), followed by *matK* (82%) and *rbcL* (70%), while ITS2 exhibited the lowest rate (67%). In total, 123 sequences generated in this work were deposited to GenBank (Accessions, ITS2: KY829137-KY829162; *matK*: KY829163-KY829195; *ndhF-rpl32*:KY829196-KY829232; *rbcL*: KY829233-KY829259) (Supplementary Table S1).

The features of the four DNA barcodes were shown in Table 1. The length of the aligned *rbcL* sequences was 350 bp with 47 variable sites and 15 informative sites. The aligned *matK* sequence was 239 bp long, with 10 variable sites and 10 informative sites. In the ITS2, the sequence was 234 bp in length, with 75 variable sites, 69 informative sites and 16 indels. For the sequence of *ndhF-rpl32*, the aligned length was 173 bp, with 14 variable sites, 12 informative sites and six indels. Among the four DNA barcodes, ITS2 had the highest proportion of variable (32.05%) and informative (29.49%) sites, followed by *rbcL* (13.43% and 4.29%) and *ndhF-rpl32* (8.09% and 6.94%), with *matK* showing the lowest values (4.18% and 4.18%).

**Table 1:** The characteristics of the four DNA barcode loci.

DNA marker	Recovery rate (%)	Sequence length (bp)	Aligned sequence length (bp)	G + C ratio (%)	No. variable sites (%)	No. parsimony informative sites (%)	Indel length (bp)
ITS2	66.67	219–224	234	66.1	75 (32.05)	69 (29.49)	16
*matK*	82.05	273	239	36.0	10 (4.18)	10 (4.18)	0
*ndhF-rpl32*	89.74	167–173	173	26.6	14 (8.09)	12 (6.94)	6
*rbcL*	69.67	485	350	40.6	47 (13.43)	15 (4.29)	0

The pairwise intraspecific distances for the barcodes ranged from a minimum value of 0.0000 for all four barcodes to a maximum value of 0.0962 (ITS2), and the mean intraspecific distances ranged from 0.0026 (*matK*) to 0.0200 (ITS2). The pairwise interspecific distances for the barcodes ranged from 0.0000 for all four barcodes to 0.1681 (ITS2), and the mean interspecific distances ranged from 0.0073 (*rbcL*) to 0.0800 (ITS2) (Table 2). ITS2 shows the highest mean intra- and inter-specific distances. The pairwise intraspecific distances for combined barcodes ranged from 0.0000 for all combinations to 0.0638 (*ndhF-rpl32* + *rbcL*), and the mean intraspecific distances ranged from 0.0027 (*matK* + *ndhF-rpl32* + ITS2) to 0.0091 (*ndhF-rpl32* + *rbcL*). The pairwise interspecific distances for combined barcodes ranged from 0.0000 (*matK* + *ndhF-rpl32*, *matK* + *rbcL* and *ndhF-rpl32* + *rbcL*) to 0.0954 (*rbcL* + ITS2) and the mean interspecific distances ranged from 0.0133 (*matK* + *rbcL*) to 0.0524 (*ndhF-rpl32* + ITS2).

**Table 2:** Genetic distance generated using Kimura 2-parameter model analysis for the candidate barcode loci and their combinations.

Barcode loci and combinations	Intraspecific distance			Interspecific distance		
	Minimum	Maximum	Mean	Minimum	Maximum	Mean
a) ITS2	0.0000	0.0962	0.0200	0.0000	0.1681	0.0800
b) *matK*	0.0000	0.0293	0.0026	0.0000	0.0335	0.0099
c) *ndhF-rpl32*	0.0000	0.0226	0.0045	0.0000	0.0292	0.0091
d) *rbcL*	0.0000	0.0914	0.0063	0.0000	0.0943	0.0073
e) *matK* + ITS2	0.0000	0.0101	0.0030	0.0084	0.0671	0.0433
f) *matK* + *ndhF-rpl32*	0.0000	0.0122	0.0015	0.0000	0.0146	0.0083
g) *matK* + *rbcL*	0.0000	0.0578	0.0060	0.0000	0.0608	0.0133
h) *ndhF-rpl32* + ITS2	0.0000	0.0131	0.0035	0.0019	0.0824	0.0524
i) *ndhF-rpl32* + *rbcL*	0.0000	0.0638	0.0091	0.0000	0.0755	0.0173
j) *rbcL* + ITS2	0.0000	0.0464	0.0067	0.0013	0.0954	0.0410
k) *matK* + *ndhF-rpl32* + ITS2	0.0000	0.0091	0.0027	0.0065	0.0582	0.0392
l) *matK* + *ndhF-rpl32* + *rbcL*	0.0000	0.0467	0.0058	0.0012	0.0551	0.0159
m) *matK* + *rbcL* + ITS2	0.0000	0.0374	0.0053	0.0049	0.0739	0.0335
n) *ndhF-rpl32* + *rbcL* + ITS2	0.0000	0.0388	0.0059	0.0010	0.0787	0.0383
o)*matK* + *ndhF-rpl32* + *rbcL* + ITS2	0.0000	0.0327	0.0064	0.0050	0.0638	0.0340

### DNA Barcoding Gap Assessment

Barcoding gaps, the absence of overlapping regions between intra- and interspecific distances, were evaluated by the results of the distribution graph obtained in the “pairwise summary” function in TaxonDNA (Supplementary Figure S1). In the study, no single- or multi-barcodes exhibited clear barcoding gaps; all barcodes overlapped between the intra- and interspecific distances. However, the mean interspecific divergence was higher than that of the corresponding intraspecific variation for each of the barcodes (Table 2). Among the single barcodes, ITS2 had the highest variation in interspecific divergence compared to the range of intraspecific distances (Table 2). When barcodes were individually analyzed, ITS2 presented the best barcode gap performance, with 69.6% of pairwise interspecific distances greater than 0.05 and 95.9% of pairwise intraspecific distances lower than 0.05. Conversely, unsatisfactory results were observed for *matK*, *ndhF-rpl32* and *rbcL* separately, with almost total overlap of intra- and interspecific variation (Supplementary Figure S1) for each.

As for the barcode combinations, the best results were found for *matK* + ITS2 and *matK* + *ndhF-rpl32* + ITS2, with 98.9% and 95.1% of pairwise interspecific distances greater than 0.05, respectively, and 91.8% of pairwise intraspecific distances lower than 0.05, both of which also outperformed any single barcode. All other barcode combinations showed clear overlap (Supplementary Figure S1).

### Species Discrimination based on TaxonDNA and Tree-based Analysis

**Figure 3:**
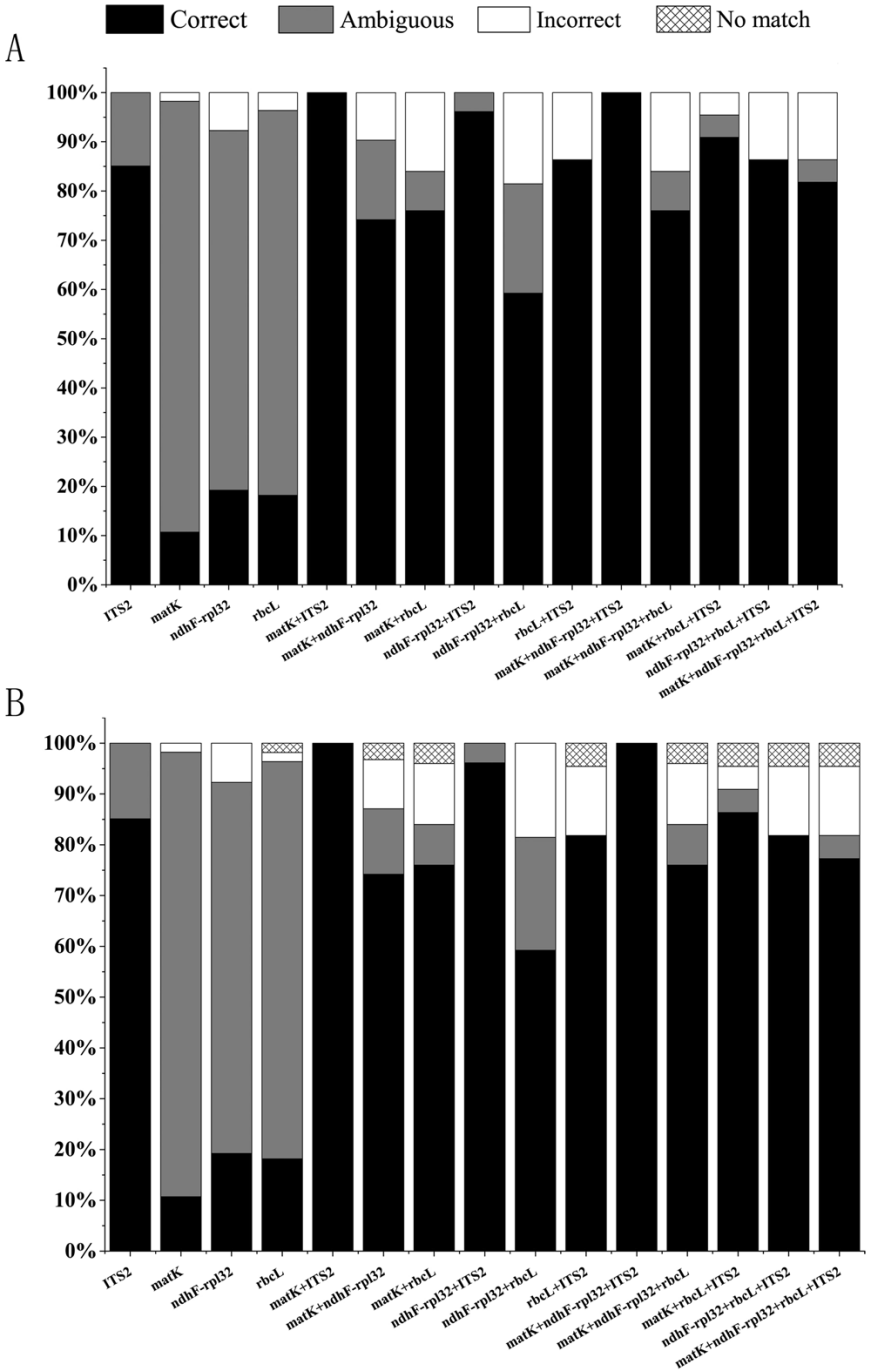
Success of species identification based on analysis of the “best match” (**A**) and “best close match” (**B**) functions of TaxonDNA program for the four DNA barcodes and their combinations.

The parameters “best match” and “best close match” from Taxon DNA were used to analyze all sequences generated in this study as well as those downloaded from the GenBank database (Fig. 3). For single-locus barcodes, both the “best match” and “best close match” methods provided the similar species discrimination success rate. ITS2 showed the highest success rate (85.1%), followed by *ndhF-rpl32* (20.0%), *rbcL* (18.2%), while *matK* exhibited the lowest rate (10.7%). The identification success rates for all barcode combinations were generally higher than those of the single barcodes. The highest success rate (100%) of barcode combinations based on the “best match” and “best close match” analysis was obtained by the two-barcode combination of *matK* + ITS2 and three-barcode combination of *matK* + *ndhF-rpl32* + ITS2. The *ndhF-rpl32* + *rbcL* combination exhibited the lowest performance for correct identification. All barcode combinations that included ITS2, i.e. *matK* + ITS2, *ndhF-rpl32* + ITS2, *rbcL* + ITS2, *matK* + *ndhF-rpl32* + ITS2, *matK* + *rbcL* + ITS2, *ndhF-rpl32* + *rbcL* + ITS2 and *matK* + *ndhF-rpl32* + *rbcL* + ITS2, provided higher identification success rates than other chloroplast DNA barcode combinations (Fig. 3).

**Figure 4:**
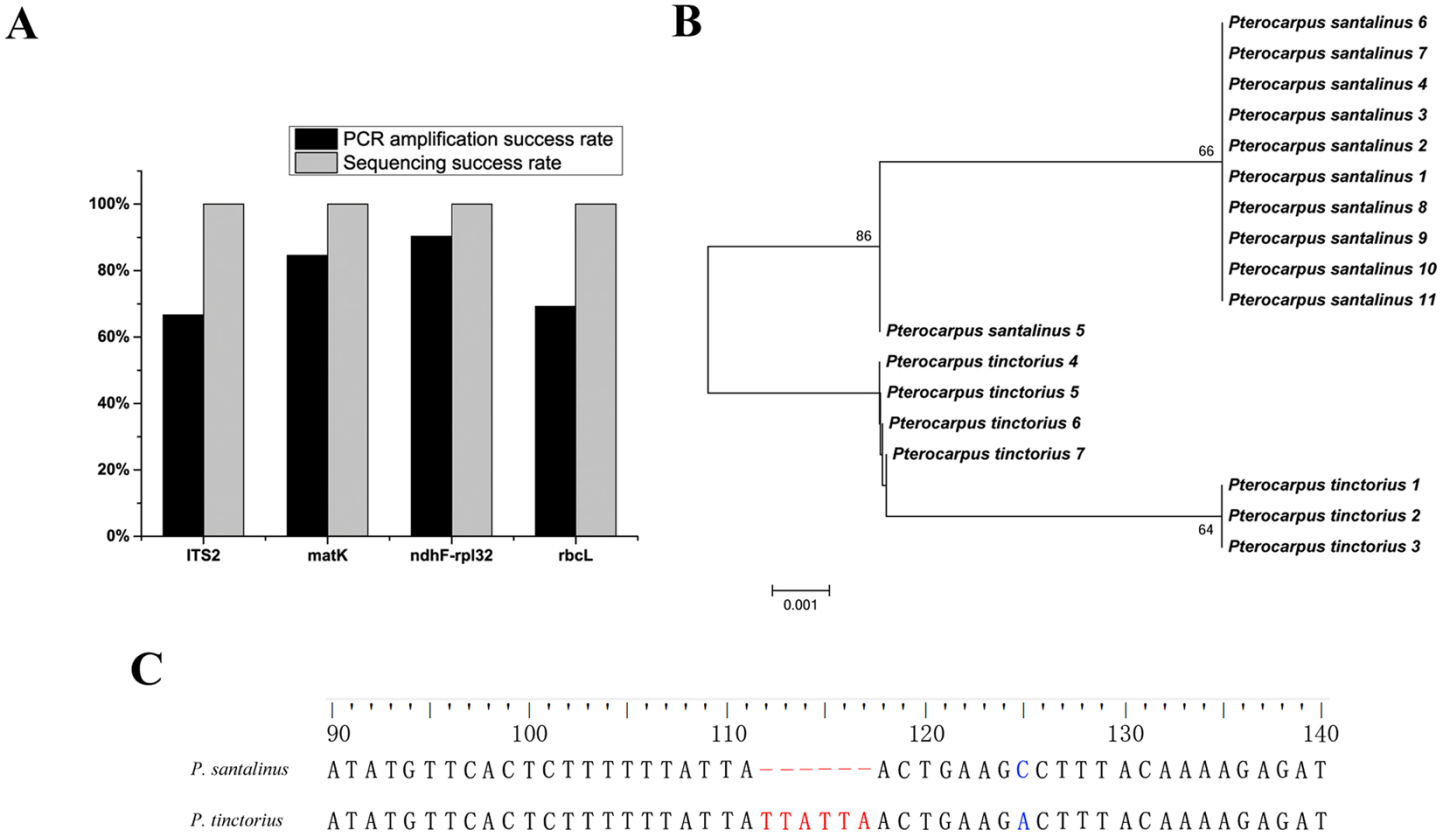
Analysis of discrimination ability of *P. santalinus* and *P. tinctorius* based on the specific mini-barcode *ndhF-rpl32*. (**A**) PCR amplification and sequencing success rate of the four DNA barcodes, (**B**) neighbor-joining tree constructed based on the barcode *ndhF-rpl32*, (**C**) variable sites of the barcode *ndhF-rpl32* between the two species.

**Figure 5:**
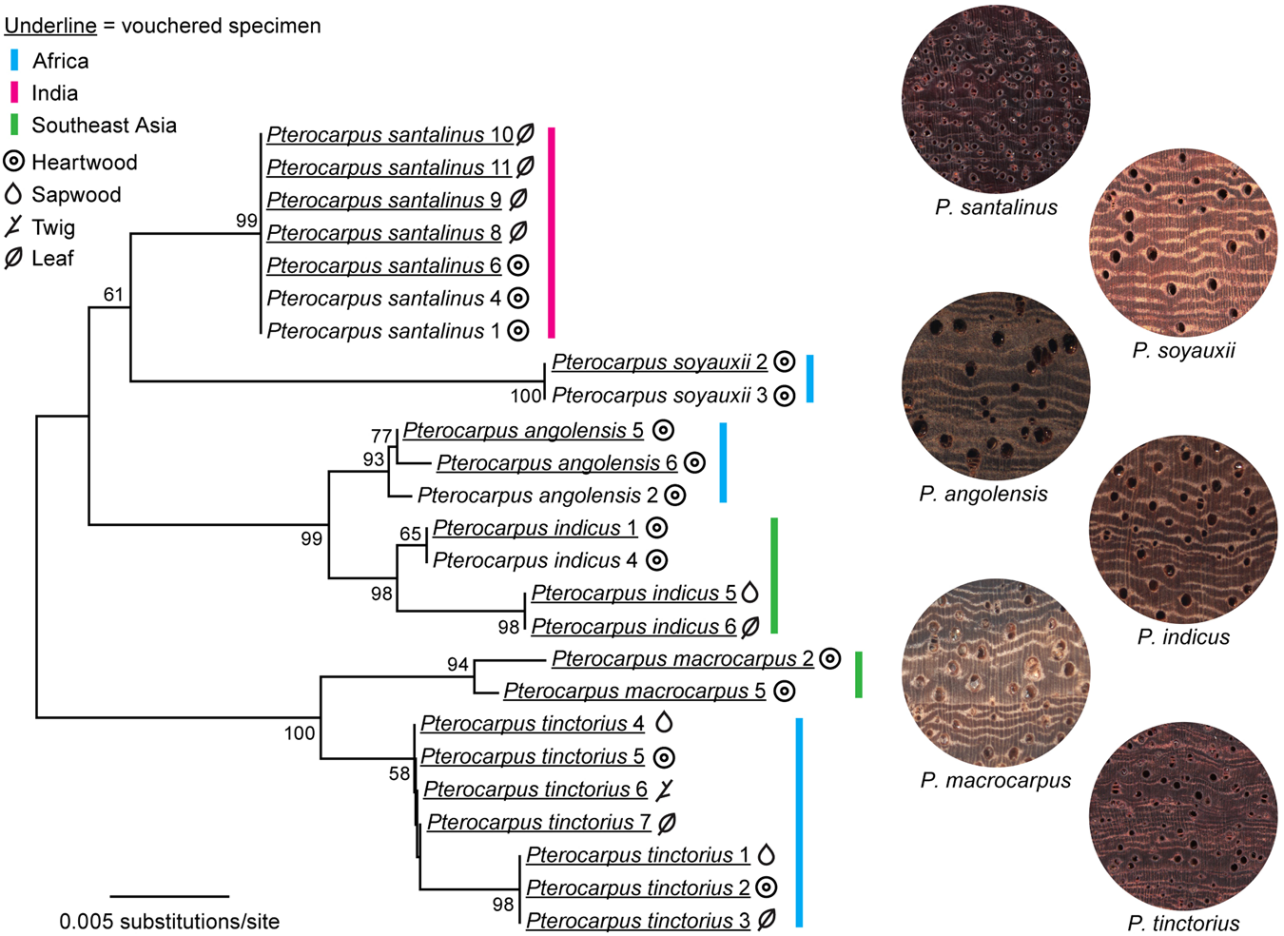
Taxon identification tree constructed using neighbor-joining analysis of P-distance showing of the best-performing barcode combination *mat*K + *ndhF-rpl32* + ITS2. Bootstrap values (>50%) are shown above the relevant branches. Photomacrographs (×16) of *Pterocarpus* xylarium specimens.

Bootstrap support for species-specific clusters based on unrooted neighbor-joining (NJ) trees for the four barcodes and their combinations were calculated (Supplementary Figure S2). When barcodes were individually analyzed, the highest species discrimination successes were obtained by ITS2 and *rbcL* (16.7%), whereas the barcodes *matK* and *ndhF-rpl32* could not distinguish any *Pterocarpus* species (Supplementary Table S4). The mini-barcode *ndhF-rpl32*, 167–173 bps in length, can separate the two anatomically similar species, *P. santalinus* and *P. tinctorius* using neighbor-joining tree analysis (Fig. 4B). Furthermore, six continuous diagnostic characters (insertion/deletion) at nucleotide positions from 112 to 117 (TTATTA) were found within the *ndhF-rpl32* region (Fig. 4C), which was a distinguishing feature based on the character-based approach. Discrimination of all six species using only one barcode was insufficient to provide an accurate resolution among the *Pterocarpus* species studied here. When combining two to four barcodes, the highest discrimination rate (100%) was obtained by *matK* + *ndhF-rpl32* + ITS2 and *matK* + *rbcL* + ITS2 (Fig. 5). Moreover, the barcode combinations that included ITS2 yielded higher success rates than other chloroplast DNA barcode combinations (Supplementary Table S4).

## Discussion

### Assessment of DNA Barcodes for *Pterocarpus*

An ideal DNA barcode should be short making it easy for recovery, and have sufficient information to provide maximal species discrimination^30–32^. While this is true for any barcode as a general principle, it is a key concern for barcodes for wood identification, because wood is a DNA-poor botanical material in the living tree, and the quality and quantity of DNA in wood degrades with industrial processing, necessitating barcodes known to be recoverable from dry wood. In these *Pterocarpus*, shorter amplicons showed a generally higher recovery rate than longer ones, with the shortest fragment *ndhF-rpl32* having the highest success rate, which is in line with several previous studies^22,28,33^. We expect that the DNA in xylarium wood specimens is typically highly fragmented^33,34^. Additionally, the nuclear ribosomal DNA region ITS2 yielded lower recovery success rate (67%) compared to the chloroplast DNA regions although it is present in multiple copies in the genome. In spite of some amplification disadvantages, ITS2 provided the best discrimination performance among the four barcodes. The superior identification power of nuclear DNA region ITS2 over plastid barcodes is also consistent with the results of other previous studies^15,19,35–37^.

Although the chloroplast DNA regions *rbcL* and *matK* were proposed as core barcodes for seed plants^31^, the two regions gave low species resolution for *Pterocarpus* in this study. Both *rbcL* and *matK* are widely used in phylogenetic analyses with over 130,000 sequences available in Genbank. Kress *et al*.^30^ showed that the *rbcL* sequence evolves slowly and this barcode has been recognized as the lowest divergence of studied plastid genes in flowering plants^30^. Consequently, on average it is not likely to be useful for identification at the species level^15,31,38–40^. It is reported that *matK* shows different discrimination success rates when it comes to different taxonomic groups (e.g. discriminating more than 90% of species in the Orchidaceae^41^) but less than 49% of species in the nutmeg family^40,42^. Meanwhile, despite its power in phylogenetic studies of other species^17,18^, *ndhF-rpl32* showed low resolution for distinguishing all six *Pterocarpus* species in this study.

No single barcode was found to be able to distinguish all six *Pterocarpus* species in this study. Overall, combined barcodes provided higher species resolution than any single barcode, which was consistent with previous studies^12,43,44^. The CBOL Plant Working Group recommended the combination barcode of *rbcL* + *matK* as the core barcode for land plants. Yan *et al*.^32^ also demonstrated that the three barcode combination of ITS + *psbA-trnH* + *matK* could give better discrimination performance than single barcodes, and was the best choice for the genus of *Rhododendron*^32^. In this study of *Pterocarpus*, the highest success rate of barcode combinations based on the “best match” and “best close match” analysis of TaxonDNA was obtained by *matK* + ITS2 and *matK* + *ndhF-rpl32* + ITS2. When the tree-based analyses (NJ) were conducted, the combination *matK* + *ndhF-rpl32* + ITS2 and *matK* + *rbcL* + ITS2 gave the best results. We conclude that the combination *matK* + *ndhF-rpl32* + ITS using these two methods is the best combination DNA barcode to resolve six of *Pterocarpus* species (Figs 3 and 5).

**Figure 6:**
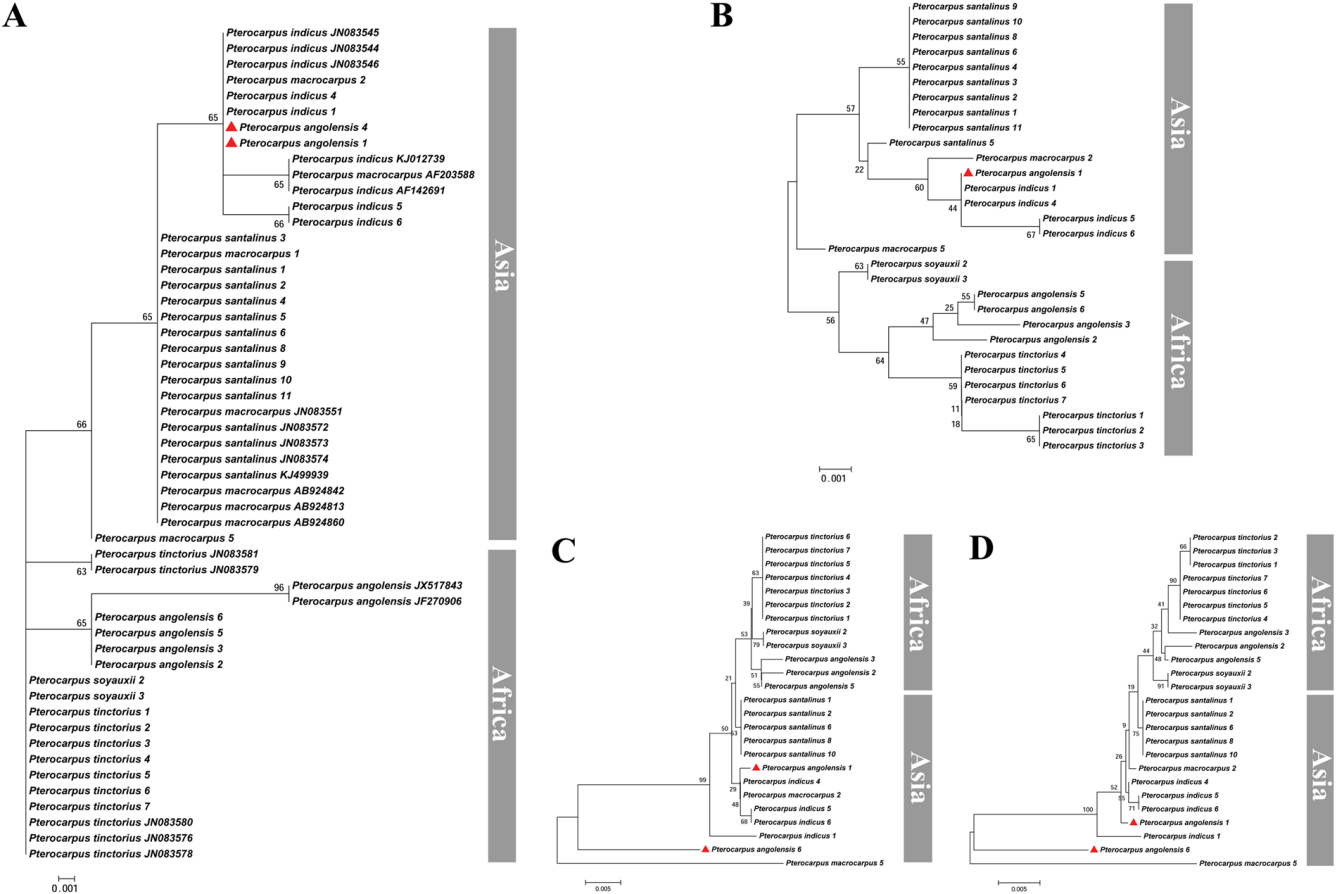
Neighbor-joining tree constructed using the barcode *matK* individual and combined showing geographic clustering pattern in *Pterocarpus* species. (**A**) *matK*, (**B**) *matK* + *ndhF-rpl32*, (**C**) *matK* + *rbcL*, (**D**) *matK* + *ndhF-rpl32* + *rbcL*.

Although the barcode *matK* individually or in combination with other chloroplast DNA barcodes yielded a low success rate for species discrimination, interestingly it has the ability to cluster studied *Pterocarpus* species according to their broad geographic origins (Fig. 6). We found that Asian and African species clustered together except for 1 or 2 samples of P. *angolensis* (Fig. 6). Here we suggest the two chloroplast locus combination of *matK* + *ndhF-rpl32* as a potential barcode for geographic origin tracking of *Pterocarpus* species when the recovery success rate is considered. It has been reported that the chloroplast DNA barcodes that are variable enough to reveal geographic structure could be used to differentiate the origin of timber^45–47^. Additionally, Lee *et al*.^12^ also showed that the DNA barcode combination *matK* + *trnL-trnF* + ITS2 had the ability of geographic clustering for *Aquilaria* species^12^.

### Species Discrimination between *P. santalinus* and *P. tinctorius* based on the Special Mini-barcode

Inasmuch as *P. santalinus* and *P. tinctorius* cannot be separated by wood anatomy but are mixed in trade, an effective method to separate these woods is critically needed. A single DNA barcode targeted to this question alone would be an effective tool, especially if the barcode were easily recovered from both species. The DNA mini-barcode *ndhF-rpl32* could give good performance for distinguishing the two closely related *Pterocarpus* species.

DNA mini-barcodes, short DNA sequences of 100–250 bp, are suitable for species identification within a given taxonomic group of old herbarium/museum specimens when high-quality DNA is not available and seriously degraded DNA is retrieved^9,48,49^. We suggest that the DNA mini-barcoding approach is suitable for species identification of woody tissues, especially in narrow cases to separate a small number of anatomically indistinguishable woods. In this study, the recovery success rate of *ndhF-rpl32* was highest among the four DNA barcodes in the study (Fig. 4) and this parameter has been used as an important criterion to determine whether DNA could be effectively isolated from wood tissues^11,22,23,34^. The reduced taxonomic discriminatory power of a mini-barcode compared to that of a full-length barcode and the taxon-specific nature of which mini-barcodes are most effective are the primary detriments of this approach. If for every group of taxa a new mini-barcode is needed, the basic principle of standardization is violated. Therefore, the choice of position of mini-barcodes from DNA genome is significant in their ability of discriminating species^48–50^. A good DNA mini-barcode candidate should be of high PCR and sequencing success without much loss of species discrimination power, and as broadly applicable as possible.

### Regarding the Utility of DNA Barcoding in the Conservation of and Controlled Trade in *Pterocarpus* Wood

Biodiversity conservation has rapidly become a focus of attention due to the sharp increase of global forest resources trade, over-exploitation and illegal logging activities. For forest protection and global trade monitoring, developing accurate species-level identification and geographic traceability for wood is a crucial and significant technical prerequisite^2^. The application of DNA barcoding to identify the species and track the geographic origin of internationally traded timber has attracted increasing interest as a potential part of global systems to support sustainable forestry and especially to reduce the behaviors of illegal logging^51^. In addition to this work, previous studies have reported the potential of DNA barcoding to support conservation efforts of wood species, e.g. *Aquilaria*^11,12^,* Dalbergia*^23,51,52^ and *Populus*^22^.

DNA barcoding can play an increased role in identification and conservation of *Pterocarpus* species, and of wood species worldwide. Availability of a reliable reference DNA barcode library remains the main obstacle of application of DNA barcoding for the next few years. Our study confirms that xylarium wood specimens are rich sources for reliable DNA sequence data. Xylarium wood specimens could certainly enhance the construction of global DNA barcode reference libraries to support species conservation worldwide, and thus continue to play a critical role as repositories of wood anatomical, chemical, and molecular information for the future.

## Materials and Methods

### Plant Materials

All wood specimens were taken from the xylarium (wood collections) of the Chinese Academy of Forestry (WOODPEDIA), the largest wood collection in China. A total of 39 specimens of 6 species of* Pterocarpus* were sampled. Four types of specimens, i.e., heartwood, sapwood, twig, and silica gel-dried leaf were collected in this study. 4–11 individuals per species were sampled. Details of the collected reference samples, including the location of vouchers, are listed in Supplementary Table S1.

### Molecular Methods

Exposed surfaces of xylarium wood specimens were removed with a sterile scalpel to avoid external contamination. Each wood sample of 500 mg was frozen in liquid nitrogen and then ground into a fine powder in a 6770 Freezer/Mill (SpexSamplePrep, Metuchen, NJ, USA).

All DNA isolations were carried out under sterile conditions. DNA from the wood specimens was extracted following the DNeasy Plant Maxi Kit (Qiagen, Hilden, Germany) protocol, modified^11^ according to Jiao *et al*.^11^. For silica gel-dried leaves, DNA was isolated using the DNeasy Plant Mini Kit (Qiagen, Hilden, Germany) following the manufacturer’s recommendations.

PCR amplification was performed in a 30 μl reaction with 15 μl of TaKaRa Premix Ex Taq (containing 0.75 units of Ex Taq DNA polymerase, 2 mM of MgCl_2_, and 200 Μm of each dNTP), 0.2 μM of each primer and approx. 10 ng of template DNA. The amplification was conducted in a Veriti 96-Well Thermal Cycler (Applied Biosystems, Foster City, CA, USA). PCR Primers and PCR cycling conditions used in this study are listed in Supplementary Table S2. The PCR products were purified using a UNIQ-10 Spin Column DNA Gel Extraction Kit (Sangon, Shanghai, China) and sequenced in both directions with the same primers used for PCR on an ABI PRISM 3730xl (Applied Biosystems, Foster City, CA, USA).

In addition to the sequences generated in this work, we downloaded sequences (from loci ITS2, *matK*, *ndhF-rpl32* and *rbcL*) (Supplementary Table S3) for specimens of *Pterocarpus* from GenBank for analysis.

### Light Microscopy

Sectioning blocks [10 mm (L) × 10 mm (R) × 10 mm (T)] were cut with razor blades and then softened in 2% ethylenediamine at 60 °C for 48 hours. Thereafter, 15 μm thick transverse, radial and tangential sections were cut on a sliding microtome. Sections were stained with a 1% aqueous safranin solution, rinsed, then mounted on glass slides and then observed under a light microscope (Olympus BX61, Japan).

### Data Analysis

Raw sequences for each region were assembled and edited using ContigExpress in Vector NTI Advance v. 10.1 (Invitrogen InforMax, Frederick, MD, USA), saved in FASTA format and deposited to GenBank (Supplementary Table S1). The edited sequences were then aligned with Clustal X 1.81^53^ followed by a manual adjustment with BioEdit software^54^. To assess the barcoding gap, the relative distribution of pairwise genetic distances was calculated using TaxonDNA^55^ under the K2P-corrected pairwise distance model^32^.

To evaluate species discrimination success, two widely used methods, TaxonDNA and a neighbor-joining tree-based approach, were applied to the four single barcode and all their possible combinations. For the TaxonDNA analysis, we used the “best match” and the “best close match” functions in the software to test the species-level discrimination rates under the K2P-corrected distance model for each barcode singly and all possible combinations of barcodes^52,56^. The “best close match” method required a threshold value which was calculated for each barcode from pairwise summary. All the results above the threshold were treated as “no match”. For the tree-based method, unrooted neighbour-joining (NJ) trees were constructed in MEGA 5^57^ with pairwise deletion and the P-distance model^32,51,58–60^. Only when all the conspecific individuals were clustered a single clade and at least one specimen in each clade was derived from a botanically vouchered collection was it considered a successful species discrimination.

## Electronic supplementary material

Supplementary information
