# Supplementary material for: DNA Barcode Authentication and Library Development for the Wood of Six Commercial Pterocarpus Species: the Critical Role of Xylarium Specimens
Source: Sci Rep. 2018 Jan 31;8:1945. doi: 10.1038/s41598-018-20381-6 (PMC5792460; doi:10.1038/s41598-018-20381-6)

DNA Barcode Authentication and Library Development for the Wood of Six Commercial *Pterocarpus* Species: the Critical Role of Xylarium Specimens

Lichao Jiao[1,2†], Min Yu[1,2†], Alex C. Wiedenhoeft[3,4,5,6], Tuo He[1,2], Jianing Li[7], Bo Liu[1,2], Xiaomei Jiang[1,2], Yafang Yin[1,2,4*]

1 Department of Wood Anatomy and Utilization, Chinese Research Institute of Wood Industry, Chinese Academy of Forestry, Beijing 100091, China

2 Wood Collections (WOODPEDIA), Chinese Academy of Forestry, Beijing 100091, China

3 Center for Wood Anatomy Research, USDA Forest Service, Forest Products Laboratory, Madison, WI 53726, USA

4 Department of Botany, University of Wisconsin, Madison, WI 53706, USA

5 Department of Forestry and Natural Resources, Purdue University, West Lafayette, IN 47907, USA

6 Ciências Biológicas (Botânica), Univesida de Estadual Paulista – Botucatu, Botucatu, São Paulo, Brasil

7 Rubber Research Institute, Chinese Academy of Tropical Agricultural Science, Hainan 571737, China

*__Corresponding Author:__ Yafang Yin, tel: +86 10 6288 9468, fax: +86 10 6288 1937; e-mail: yafang@caf.ac.cn

† These authors contributed to the work equally and should be regarded as co-first authors.

Table S1 Samples details and sequences information in this study.

Code	Species	Sample ID	Voucher ID	Abbreviation	Type of sample	Sample notes	Region of origin	GenBank accession numbers			
								ITS2	*matK*	*ndhF-rpl32*	*rbcL*
1		CAFW22639	-	*P. angolensis* 1	Heartwood		South Africa	—	KY829163	KY829196	KY829233
2		CAFW23604	-	*P. angolensis* 2	Heartwood		Africa	KY829137	KY829164	KY829197	KY829234
3	*Pterocarpus angolensis* DC.	CAFW7539	-	*P. angolensis* 3	Heartwood		East Africa	—	KY829165	KY829198	KY829235
4		CAFW15540	CRIWI-69355	*P. angolensis* 4	Heartwood	Collected by Collection Team of Chinese Research Institute of Wood Industry, 1969; identified by J	Africa	—	KY829166	—	—
5		CAFW17977	CRIWI-81257	*P. angolensis* 5	Heartwood	Collected by Collection Team of Chinese Research Institute of Wood Industry, 1981; identified by J	Africa	KY829138	KY829167	KY829199	KY829236
6		CAFW18219	H-82398	*P. angolensis* 6	Heartwood	Collected by Hamblin, 1982; identified by J Cheng	Zimbabwe	KY829139	KY829168	KY829200	KY829237
7		CAFW20667	CRIWI-88320	*P.indicus* 1	Heartwood	Collected by Collection Team of Chinese Research Institute of Wood Industry, 1988; identified by	Malaysia	KY829140	KY829169	KY829201	KY829238
8		CAFW22939	-	*P.indicus 2*	Heartwood		Vietnam	—	—	—	—
9	*Pterocarpus indicus* Willd.	CAFW22940	-	*P.indicus* 3	Heartwood		Vietnam	—	—	KY829203	—
10		CAFW23342	-	*P.indicus* 4	Heartwood		Indonesia	KY829141	KY829171	KY829204	KY829239
11		W001	JLC-14331	*P.indicus 5*	Sapwood	Collected by L Jiao, Novermber 10th, 2014; identified by X Jiang	China	KY829142	KY829172	KY829205	KY829240
12		L001	JLC-14264	*P.indicus* 6	Leaf	Collected by L Jiao, Novermber 10th, 2014; identified by X Jiang	China	KY829143	KY829173	KY829206	KY829241
13		CAFW20336	CRIWI-87241	*P. macrocarpus* 1	Heartwood	Collected by Collection Team of Chinese Research Institute of Wood Industry, 1987; identified by J	Thailand	—	KY829174	—	—
14		CAFW19684	LXX-84388	*P. macrocarpus* 2	Heartwood	Collected by X Lu, 1984; identified by J Cheng	Myanmar	KY829144	KY829175	KY829207	KY829242
15	*Pterocarpus macrocarpus* Kurz	CAFW15231	CJB-69249	*P. macrocarpus* 3	Heartwood	Collected by J Chen, 1969; identified by J Cheng	Myanmar	—	—	KY829208	KY829243
16		CAFW9750	CRIWI-57275	*P. macrocarpus* 4	Heartwood	Collected by Collection Team of Chinese Research Institute of Wood Industry, 1957; identified by J	Cambodia	—	—	KY829209	KY829244
17		W002	LB-15396	*P. macrocarpus* 5	Heartwood	Collected by B Liu, 2015; identified by X Jiang	Myanmar	KY829145	KY829176	KY829210	KY829245
18		CAFW23676	-	*P. santalinus* 1	Heartwood		India	KY829146	KY829177	KY829211	KY829246
19		CAFW23675	-	*P. santalinus* 2	Heartwood		India	—	KY829178	KY829212	KY829247
20		CAFW23674	-	*P. santalinus* 3	Heartwood		India	—	KY829179	KY829213	—
21		CAFW22653	-	*P. santalinus* 4	Heartwood		India	KY829147	KY829180	KY829214	—
22		W003	LB-15309	*P. santalinus* 5	Sapwood	Collected by B Liu, 2015; identified by X Jiang	India	—	KY829181	KY829215	—
23	*Pterocarpus santalinus* L.f.	W004	LB-15360	*P. santalinus* 6	Heartwood	Collected by B Liu, 2015; identified by X Jiang	India	KY829148	KY829182	KY829216	KY829248
24		L002	LQQ-16266	*P. santalinus* 7	Leaf	Collected by Q Li, Auguest 24th, 2016; identified by X Jiang	China	KY829149	—	KY829217	—
25		L003	LQQ-16267	*P. santalinus* 8	Leaf	Collected by Q Li, Auguest 24th, 2016; identified by X Jiang	China	KY829150	KY829183	KY829218	KY829249
26		L004	LQQ-16268	*P. santalinus* 9	Leaf	Collected by Q Li, Auguest 24th, 2016; identified by X Jiang	China	KY829151	KY829184	KY829219	—
27		L005	LQQ-16269	*P. santalinus* 10	Leaf	Collected by Q Li, Auguest 24th, 2016; identified by X Jiang	China	KY829152	KY829185	KY829220	KY829250
28		L006	LQQ-16270	*P. santalinus* 11	Leaf	Collected by Q Li, Auguest 24th, 2016; identified by X Jiang	China	KY829153	KY829186	KY829221	—
29		CAFW5159	-	*P. soyauxii* 1	Heartwood		West Africa	—	—	—	—
30	*Pterocarpus soyauxii* Taub.	CAFW21887	LPJXM-93277	*P. soyauxii* 2	Heartwood	Collected by P Liu X Jiang, 1993; identified by X Jiang	Cameroun	KY829154	KY829187	KY829223	KY829251
31		CAFW23477	-	*P. soyauxii* 3	Heartwood		Africa	KY829155	KY829188	KY829224	KY829252
32		CAFW15076	CRIWI-67280	*P. soyauxii* 4	Heartwood	Collected by Collection Team of Chinese Research Institute of Wood Industry, 1967; identified by J	Congo	—	—	KY829225	—
33		W005	XD-15231	*P. tinctorius* 1	Sapwood	Collected by D Xiao, April 16th, 2015; identified by X Jiang	Congo	KY829156	KY829189	KY829226	KY829253
34		W006	XD-15232	*P. tinctorius* 2	Heartwood	Collected by D Xiao, April 16th, 2015; identified by X Jiang	Congo	KY829157	KY829190	KY829227	KY829254
35		L007	XD-15233	*P. tinctorius* 3	Leaf	Collected by D Xiao, April 16th, 2015; identified by X Jiang	Congo	KY829158	KY829191	KY829228	KY829255
36	*Pterocarpus tinctorius* Welw.	W007	XCY-16325	*P. tinctorius* 4	Sapwood	Collected by C Xu, April 4th, 2016; identified by X Jiang	Congo	KY829159	KY829192	KY829229	KY829256
37		W008	XCY-16326	*P. tinctorius* 5	Heartwood	Collected by C Xu, April 4th, 2016; identified by X Jiang	Congo	KY829160	KY829193	KY829230	KY829257
38		W009	XCY-16328	*P. tinctorius* 6	Twig	Collected by C Xu, April 4th, 2016; identified by X Jiang	Congo	KY829161	KY829194	KY829231	KY829258
39		L008	XCY-16330	*P. tinctorius* 7	Leaf	Collected by C Xu, April 4th, 2016; identified by X Jiang	Congo	KY829162	KY829195	KY829232	KY829259

Note: 1) "-" of voucher ID means no available voucher record.

2) "-" of GenBank Accession numbers means failed PCR amplifications.

Table S2 Primer pairs of DNA region and reaction conditions applied for the PCR amplification tests in this study

DNA barcode	Name of Primers	Primer sequences (5′-3′)	PCR reaction conditions	References
ITS2	ITS2-F	ATGCGATACTTGGTGTGA	94°C 90s; 94°C 5s, 51°C 30s, 72°C 20s, 40 cycles; 72°C 7min	This study
	ITS2-R	TAGCCCCGCCTGACCTGA		
matK	matK-F	TTTCGTCTACCTTATCCTTCTTCA	94°C 90s; 94°C 5s, 52°C 30s, 72°C 20s, 40 cycles; 72°C 7min	This study
	matK-R	CTAGCATTTGACTCCGTACCAC		
ndhF-rpl32	NR-F	GATTCGTAATGAGTTTTGTTTC	94°C 90s; 94°C 5s, 47°C 30s, 72°C 20s, 40 cycles; 72°C 7min	This study
	NR-R	AGGAATTTGTTTATTGGACC		
rbcL	rbcL-F	TGCCGAATCTTCTACTGG	94°C 90s; 94°C 5s, 50°C 30s, 72°C 20s, 40 cycles; 72°C 7min	This study
	rbcL-R	ATAAGAAACGGTCCCTCC		

Table S3 Barcode sequences downloaded from GenBank.

DNA barcode locus	Species	GenBank accession				
ITS2	*Pterocarpus angolensis*	JN083472	JN083473			
	Pterocarpus indicus	JN083480	JN083481	JN083482	AF269177	
	Pterocarpus macrocarpus	AF269176				
	Pterocarpus santalinus	JN083516	JN083517	JN083518		
	Pterocarpus soyauxii	JN083519	JN083520			
	Pterocarpus tinctorius	JN083522	JN083523	JN083524	JN083525	JN083526
		JN083527	JN083528			
matK	*Pterocarpus angolensis*	JX517843	JF270906			
	Pterocarpus indicus	KJ012739	AF142691	JN083544	JN083545	JN083546
	Pterocarpus macrocarpus	AF203588	JN083551	AB924842	AB924813	AB924860
	Pterocarpus santalinus	JN083572	JN083573	JN083574	KJ499939	
	Pterocarpus soyauxii	—				
	Pterocarpus tinctorius	JN083576	JN083578	JN083579	JN083580	JN083581
ndhF-rpl32	*Pterocarpus angolensis*	JN083592	JN083593			
	Pterocarpus indicus	JN083599	JN083600			
	Pterocarpus macrocarpus	JN083605				
	Pterocarpus santalinus	JN083626	JN083627	JN083628		
	Pterocarpus soyauxii	—				
	Pterocarpus tinctorius	JN083631	JN083632	JN083633	JN083634	JN083635
rbcL	*Pterocarpus angolensis*	JF265564	JX572902	JN083716	JN083717	
	Pterocarpus indicus	JN083723	JN083724	JN083725	JF739158	JF739060
		JF738809	JF738773			
	Pterocarpus macrocarpus	AB925590	AB925475	AB925456	AB925424	JN083730

	Pterocarpus santalinus	KJ667606	KF381129	JN083757	JN083758	JN083759
	Pterocarpus soyauxii	—				
	Pterocarpus tinctorius	JN083762	JN083763	JN083765	JN083766	JN083767

Table S4 Discrimination success rate of *Pterocarpus* species using the neighbor-joining analysis based on the four DNA barcodes and their combinations.

Barcode loci and combinations	Identification success rate
a) ITS2	(16.67%) 1/6
b) *matK*	(0%) 0/6
c) *ndhF-rpl32*	(0%) 0/6
d) *rbcL*	(16.67%) 1/6
e) *matK* +ITS2	(66.67%) 4/6
f) *matK* +*ndhF-rpl32*	(33.33%) 2/6
g) *matK* +*rbcL*	(50%) 3/6
h) *ndhF-rpl32* +ITS2	(66.67%) 4/6
i) *ndhF-rpl32* +*rbcL*	(50%) 3/6
j) *rbcL* +ITS2	(66.67%) 4/6
k) *matK* +*ndhF-rpl32* +ITS2	(100%) 6/6
l) *matK* +*ndhF-rpl32* +*rbcL*	(50%) 3/6
m) *matK*+*rbcL*+ ITS2	(100%) 6/6
n) *ndhF-rpl32*+*rbcL*+ ITS2	(66.67%) 4/6
o) *matK*+*ndhF-rpl32*+*rbcL*+ ITS2	(66.67%) 4/6

Figure S1 Relative distributions of interspecific and intraspecific Kimura two-parameter (K2P) distances for the four DNA barcodes and their combinations.

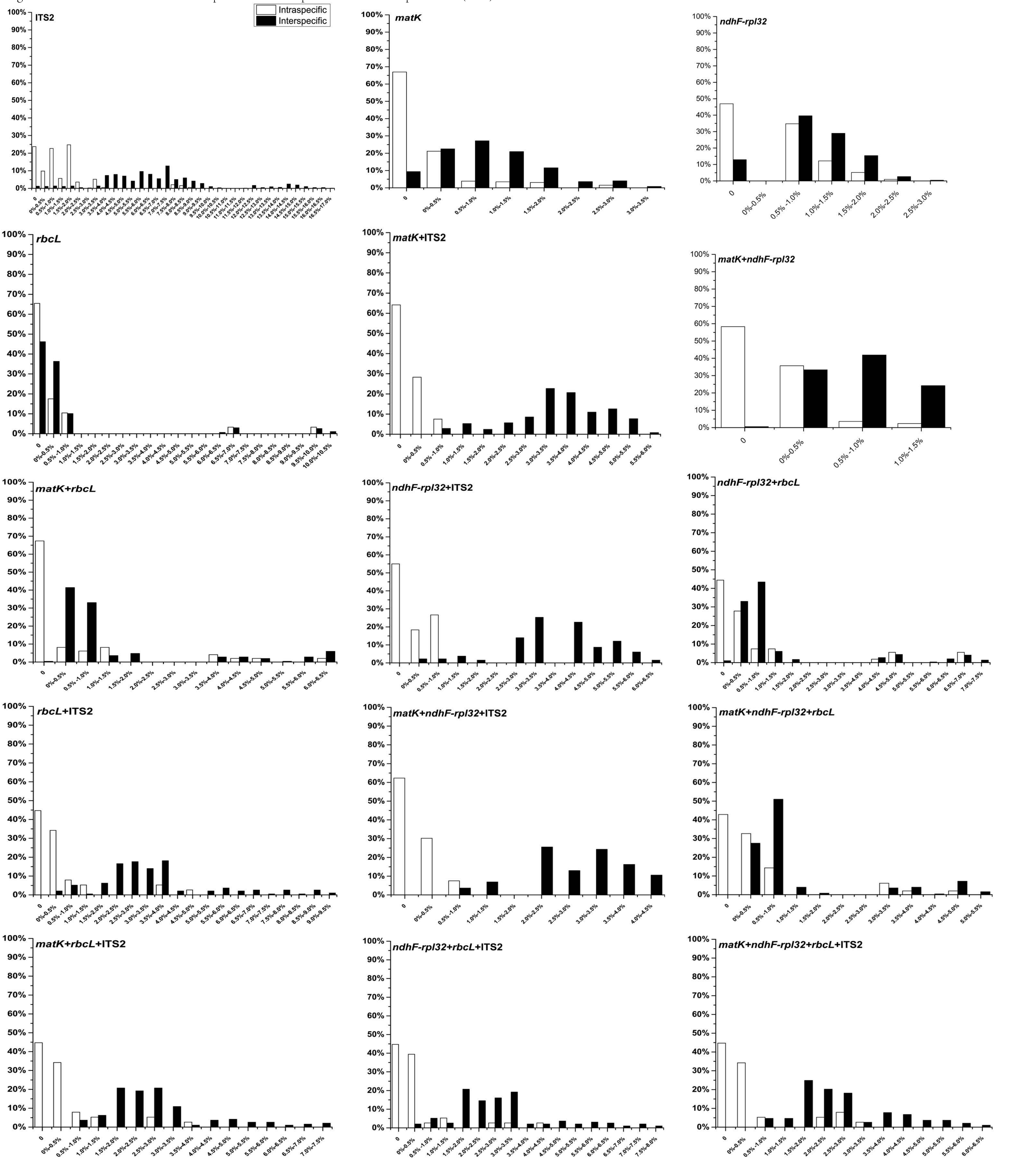

Figure S2 Taxon identification trees constructed using neighbor-joining analysis of P-distance showing patterns of the four DNA barcodes and their combinations.

a) ITS2

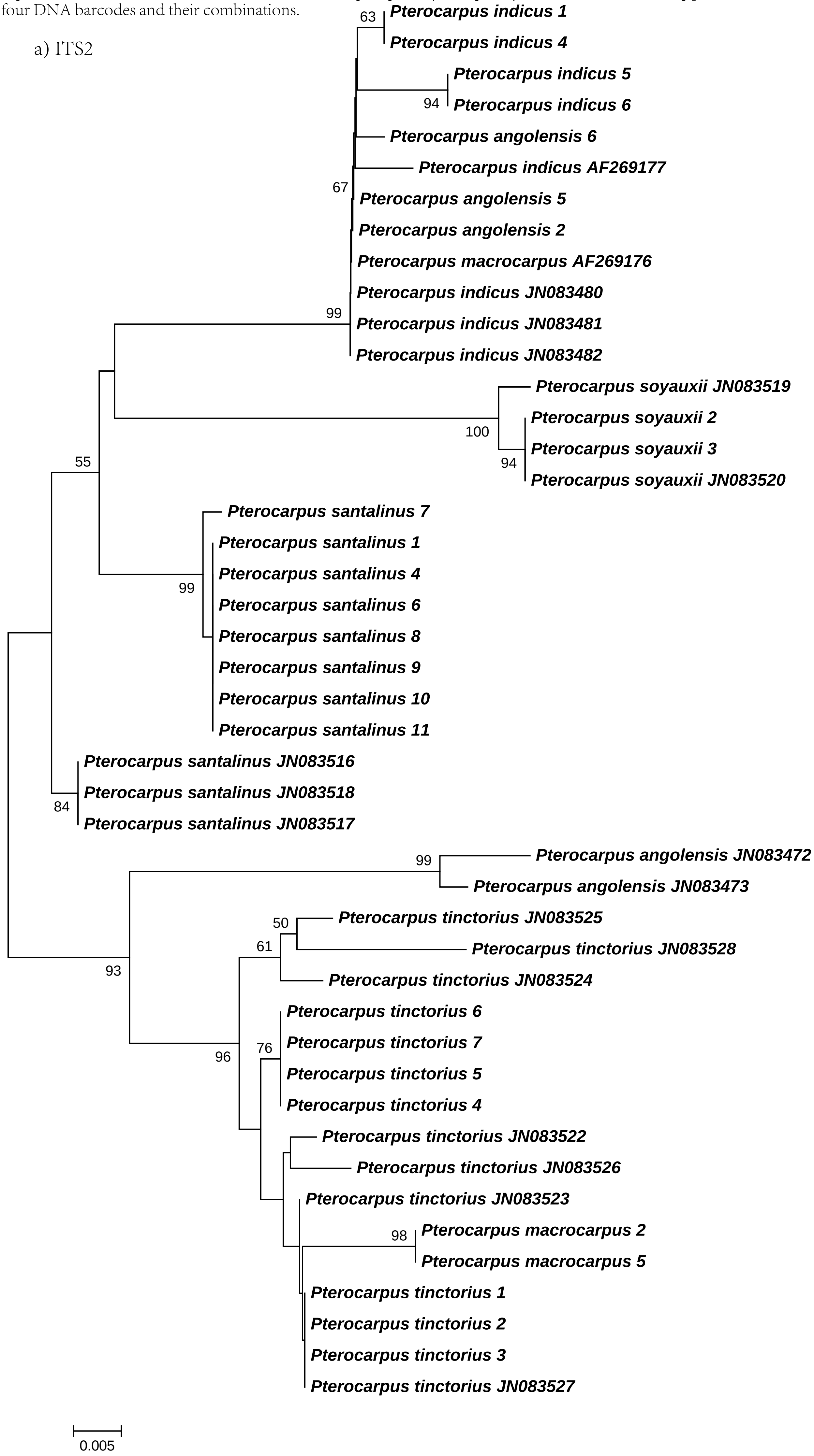

b) matK

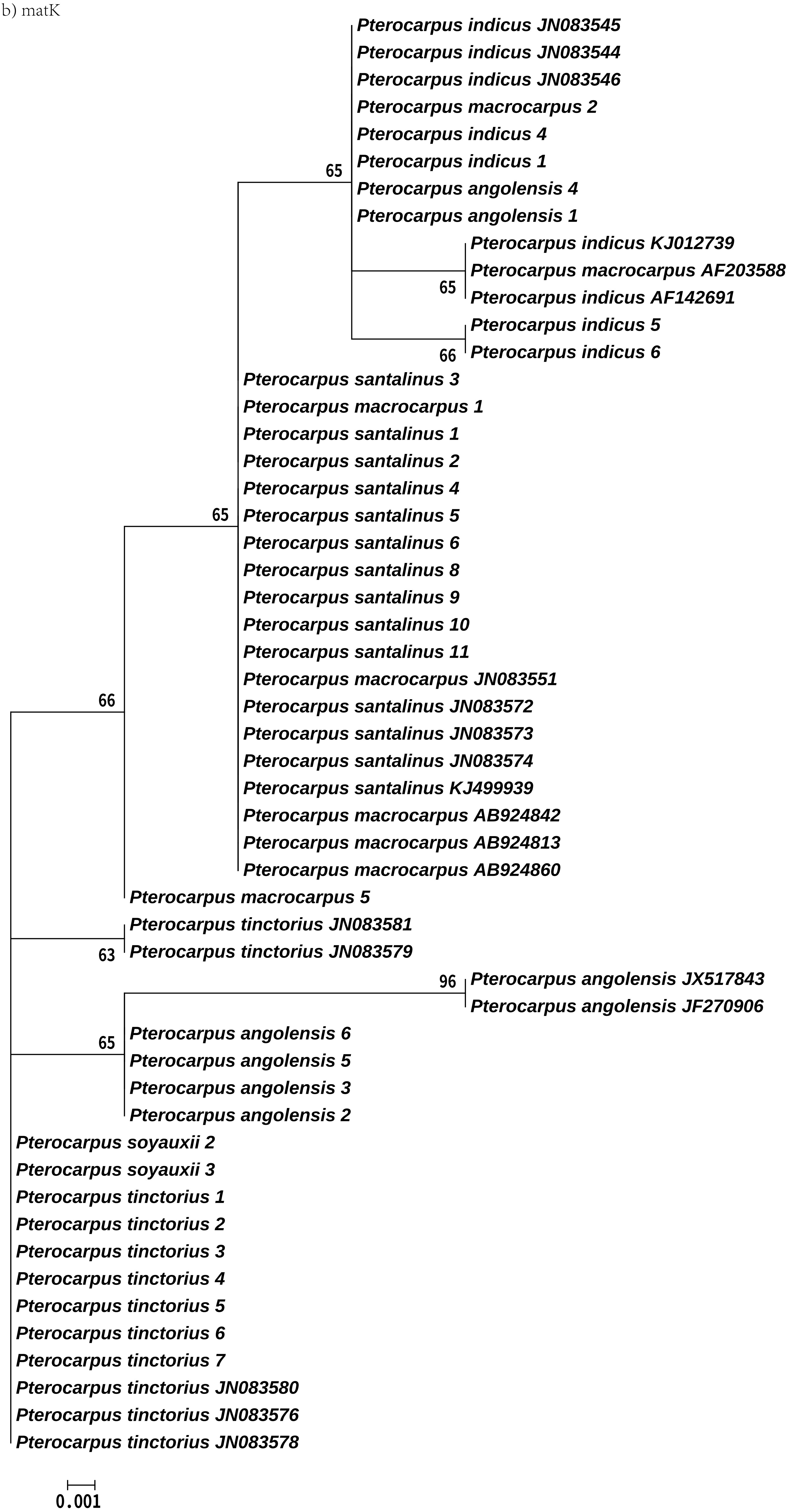
Pterocarpus indicus JN083545
Pterocarpus indicus JN083544
Pterocarpus indicus JN083546
Pterocarpus macrocarpus 2
Pterocarpus indicus 4
Pterocarpus indicus 1
Pterocarpus angolensis 4
Pterocarpus angolensis 1
Pterocarpus indicus KJ012739
Pterocarpus macrocarpus AF203588
Pterocarpus indicus AF142691
Pterocarpus indicus 5
Pterocarpus indicus 6
Pterocarpus santalinus 3
Pterocarpus macrocarpus 1
Pterocarpus santalinus 1
Pterocarpus santalinus 2
Pterocarpus santalinus 4
Pterocarpus santalinus 5
Pterocarpus santalinus 6
Pterocarpus santalinus 8
Pterocarpus santalinus 9
Pterocarpus santalinus 10
Pterocarpus santalinus 11
Pterocarpus macrocarpus JN083551
Pterocarpus santalinus JN083572
Pterocarpus santalinus JN083573
Pterocarpus santalinus JN083574
Pterocarpus santalinus KJ499939
Pterocarpus macrocarpus AB924842
Pterocarpus macrocarpus AB924813
Pterocarpus macrocarpus AB924860
Pterocarpus macrocarpus 5
Pterocarpus tinctorius JN083581
Pterocarpus tinctorius JN083579
Pterocarpus angolensis JX517843
Pterocarpus angolensis JF270906
Pterocarpus angolensis 6
Pterocarpus angolensis 5
Pterocarpus angolensis 3
Pterocarpus angolensis 2
Pterocarpus soyauxii 2
Pterocarpus soyauxii 3
Pterocarpus tinctorius 1
Pterocarpus tinctorius 2
Pterocarpus tinctorius 3
Pterocarpus tinctorius 4
Pterocarpus tinctorius 5
Pterocarpus tinctorius 6
Pterocarpus tinctorius 7
Pterocarpus tinctorius JN083580
Pterocarpus tinctorius JN083576
Pterocarpus tinctorius JN083578
65
65
66
65
66
63
96
65
0.001

c) ndhF-rpl32

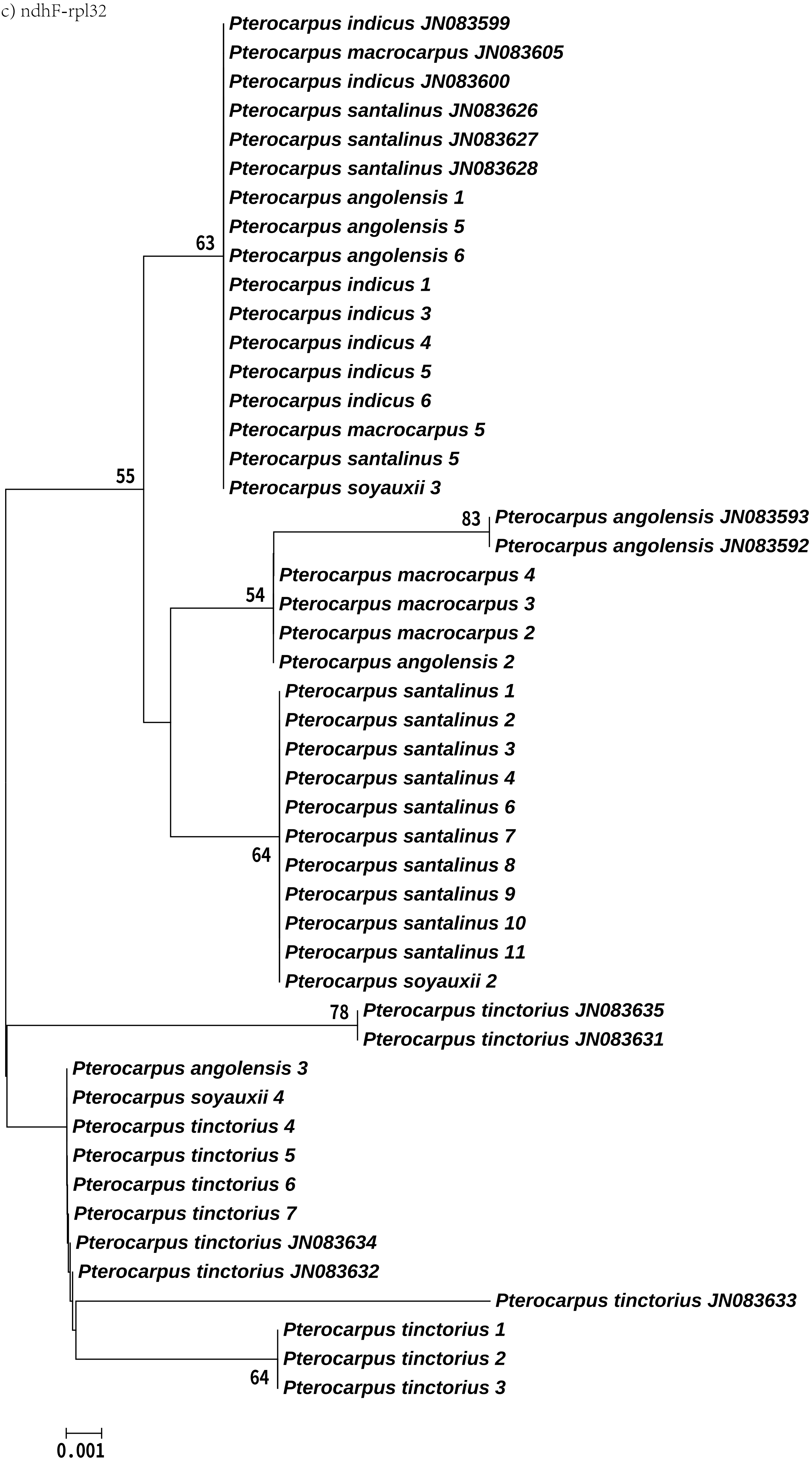
Pterocarpus indicus JN083599
Pterocarpus macrocarpus JN083605
Pterocarpus indicus JN083600
Pterocarpus santalinus JN083626
Pterocarpus santalinus JN083627
Pterocarpus santalinus JN083628
Pterocarpus angolensis 1
Pterocarpus angolensis 5
Pterocarpus angolensis 6
Pterocarpus indicus 1
Pterocarpus indicus 3
Pterocarpus indicus 4
Pterocarpus indicus 5
Pterocarpus indicus 6
Pterocarpus macrocarpus 5
Pterocarpus santalinus 5
Pterocarpus soyauxii 3
63
55
83
Pterocarpus angolensis JN083593
Pterocarpus angolensis JN083592
Pterocarpus macrocarpus 4
Pterocarpus macrocarpus 3
Pterocarpus macrocarpus 2
Pterocarpus angolensis 2
54
Pterocarpus santalinus 1
Pterocarpus santalinus 2
Pterocarpus santalinus 3
Pterocarpus santalinus 4
Pterocarpus santalinus 6
Pterocarpus santalinus 7
Pterocarpus santalinus 8
Pterocarpus santalinus 9
Pterocarpus santalinus 10
Pterocarpus santalinus 11
Pterocarpus soyauxii 2
64
78
Pterocarpus tinctorius JN083635
Pterocarpus tinctorius JN083631
Pterocarpus angolensis 3
Pterocarpus soyauxii 4
Pterocarpus tinctorius 4
Pterocarpus tinctorius 5
Pterocarpus tinctorius 6
Pterocarpus tinctorius 7
Pterocarpus tinctorius JN083634
Pterocarpus tinctorius JN083632
Pterocarpus tinctorius JN083633
Pterocarpus tinctorius 1
Pterocarpus tinctorius 2
Pterocarpus tinctorius 3
64
0.001

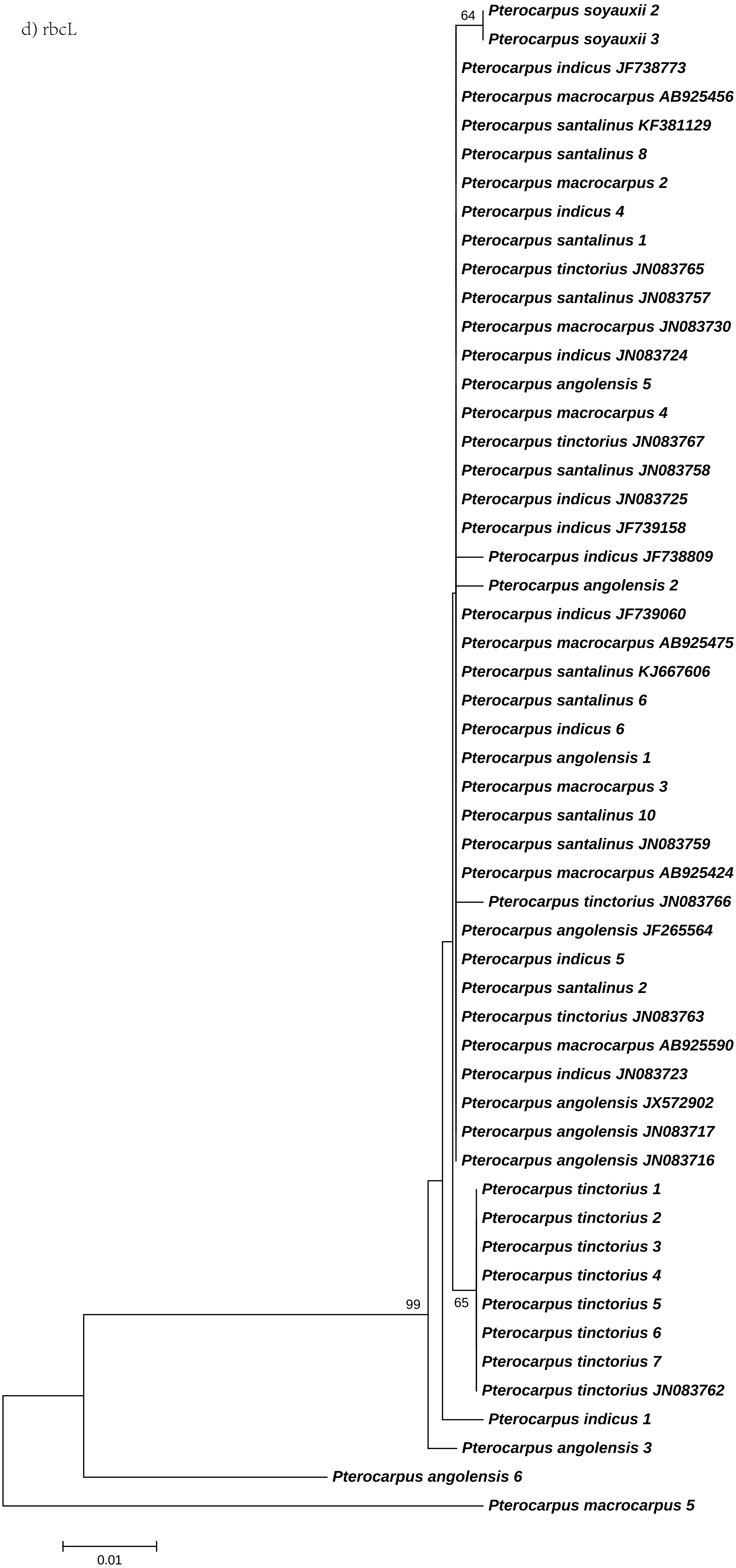
d) rbcL
64
Pterocarpus soyauxii 2
Pterocarpus soyauxii 3
Pterocarpus indicus JF738773
Pterocarpus macrocarpus AB925456
Pterocarpus santalinus KF381129
Pterocarpus santalinus 8
Pterocarpus macrocarpus 2
Pterocarpus indicus 4
Pterocarpus santalinus 1
Pterocarpus tinctorius JN083765
Pterocarpus santalinus JN083757
Pterocarpus macrocarpus JN083730
Pterocarpus indicus JN083724
Pterocarpus angolensis 5
Pterocarpus macrocarpus 4
Pterocarpus tinctorius JN083767
Pterocarpus santalinus JN083758
Pterocarpus indicus JN083725
Pterocarpus indicus JF739158
Pterocarpus indicus JF738809
Pterocarpus angolensis 2
Pterocarpus indicus JF739060
Pterocarpus macrocarpus AB925475
Pterocarpus santalinus KJ667606
Pterocarpus santalinus 6
Pterocarpus indicus 6
Pterocarpus angolensis 1
Pterocarpus macrocarpus 3
Pterocarpus santalinus 10
Pterocarpus santalinus JN083759
Pterocarpus macrocarpus AB925424
Pterocarpus tinctorius JN083766
Pterocarpus angolensis JF265564
Pterocarpus indicus 5
Pterocarpus santalinus 2
Pterocarpus tinctorius JN083763
Pterocarpus macrocarpus AB925590
Pterocarpus indicus JN083723
Pterocarpus angolensis JX572902
Pterocarpus angolensis JN083717
Pterocarpus angolensis JN083716
Pterocarpus tinctorius 1
Pterocarpus tinctorius 2
Pterocarpus tinctorius 3
Pterocarpus tinctorius 4
65
Pterocarpus tinctorius 5
Pterocarpus tinctorius 6
Pterocarpus tinctorius 7
Pterocarpus tinctorius JN083762
99
Pterocarpus indicus 1
Pterocarpus angolensis 3
Pterocarpus angolensis 6
Pterocarpus macrocarpus 5
0.01

e) matK+ITS2

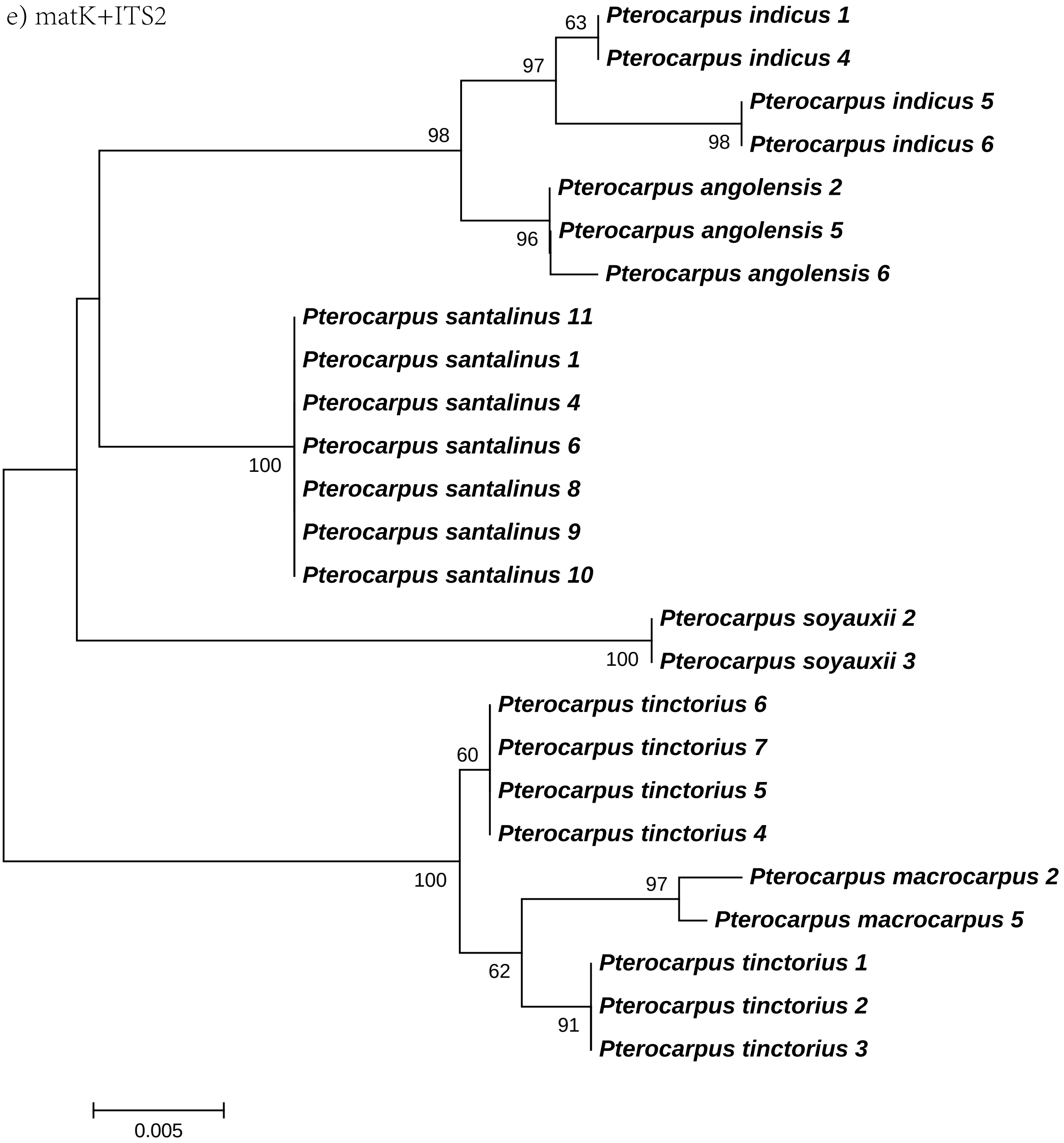

63
97
98
98
96
100
100
60
100
97
62
91
Pterocarpus indicus 1
Pterocarpus indicus 4
Pterocarpus indicus 5
Pterocarpus indicus 6
Pterocarpus angolensis 2
Pterocarpus angolensis 5
Pterocarpus angolensis 6
Pterocarpus santalinus 11
Pterocarpus santalinus 1
Pterocarpus santalinus 4
Pterocarpus santalinus 6
Pterocarpus santalinus 8
Pterocarpus santalinus 9
Pterocarpus santalinus 10
Pterocarpus soyauxii 2
Pterocarpus soyauxii 3
Pterocarpus tinctorius 6
Pterocarpus tinctorius 7
Pterocarpus tinctorius 5
Pterocarpus tinctorius 4
Pterocarpus macrocarpus 2
Pterocarpus macrocarpus 5
Pterocarpus tinctorius 1
Pterocarpus tinctorius 2
Pterocarpus tinctorius 3
0.005

f) matK+ndhF-rpl32

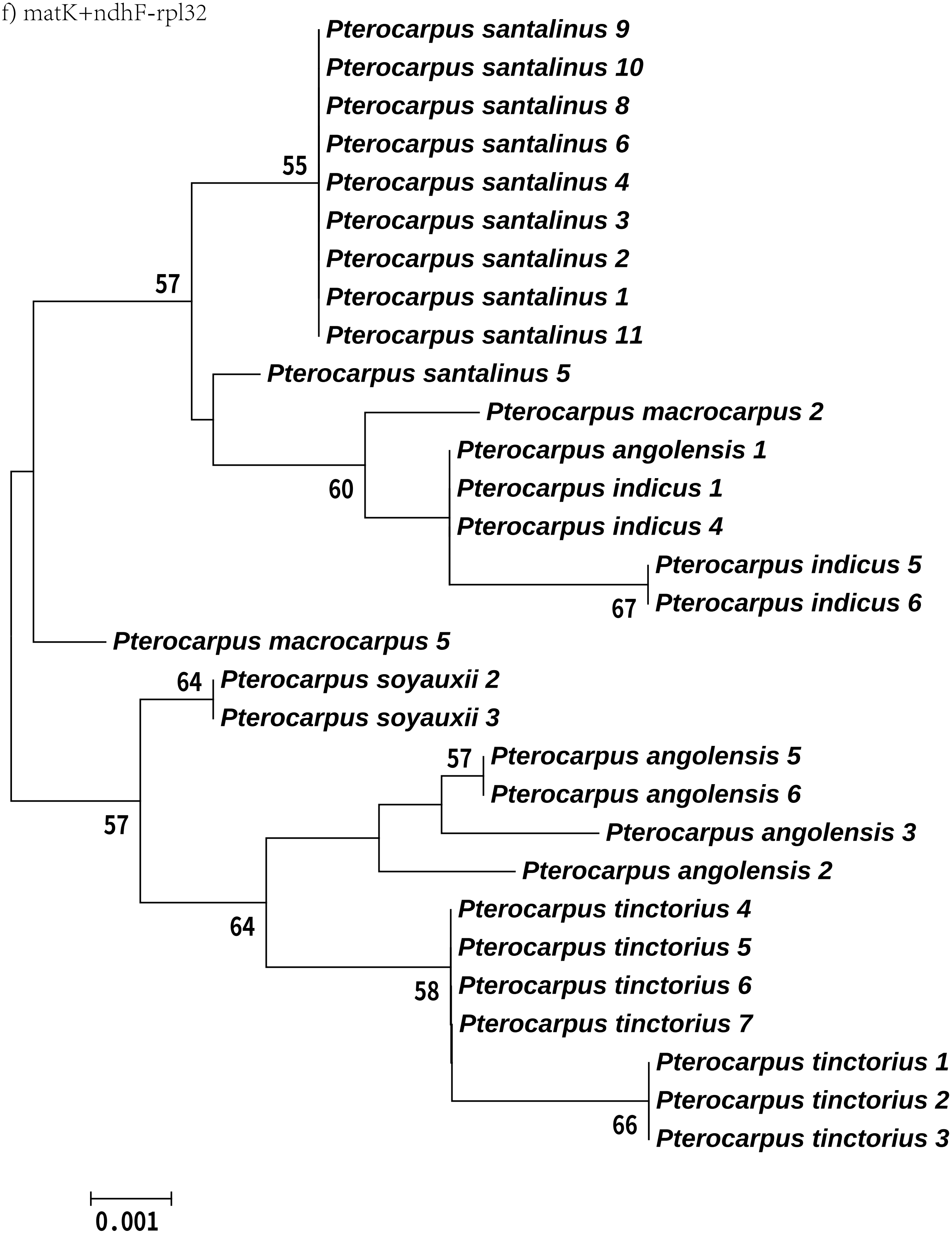

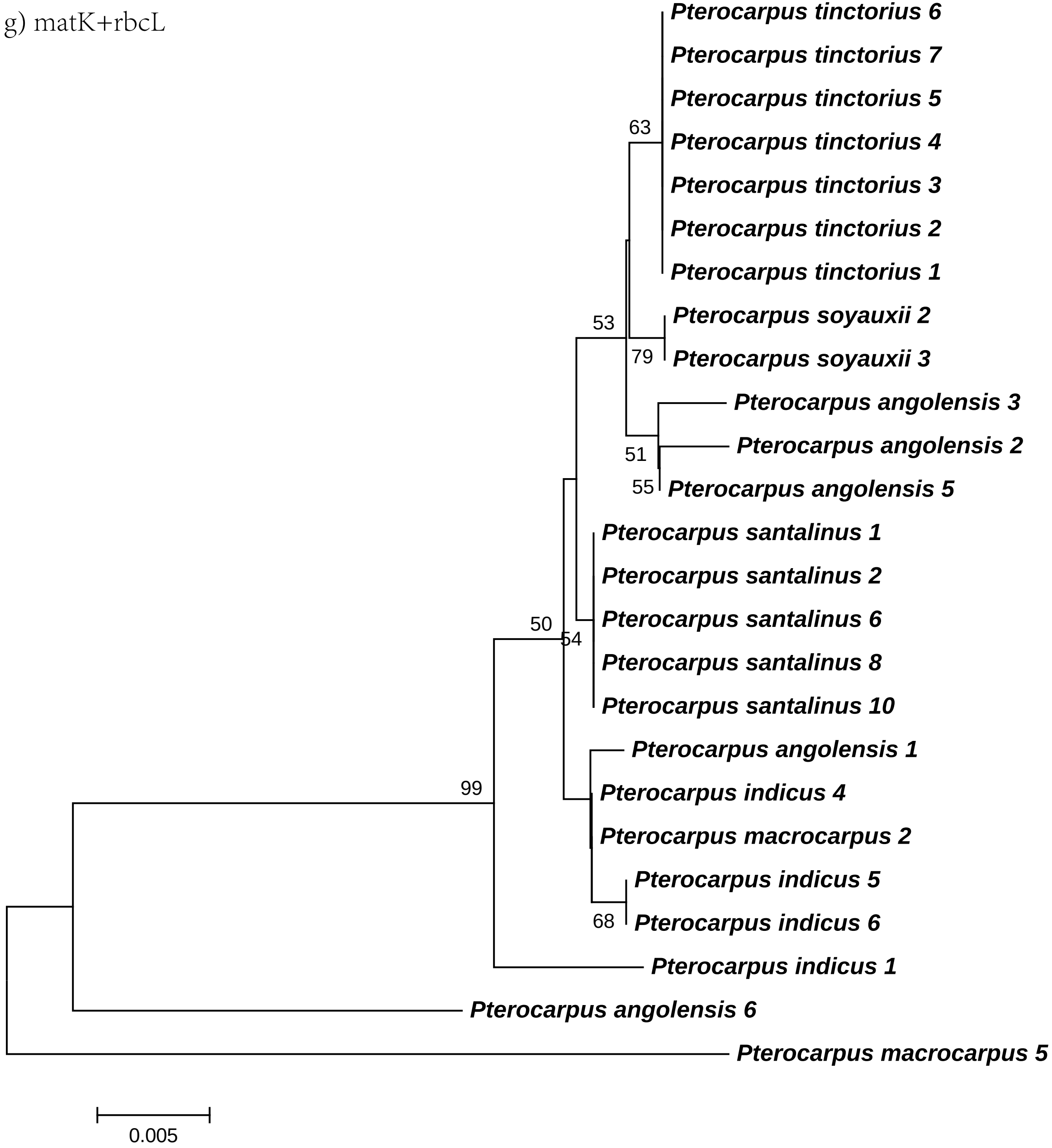
g) matK+rbcL
Pterocarpus tinctorius 6
Pterocarpus tinctorius 7
Pterocarpus tinctorius 5
Pterocarpus tinctorius 4
Pterocarpus tinctorius 3
Pterocarpus tinctorius 2
Pterocarpus tinctorius 1
Pterocarpus soyauxii 2
Pterocarpus soyauxii 3
Pterocarpus angolensis 3
Pterocarpus angolensis 2
Pterocarpus angolensis 5
Pterocarpus santalinus 1
Pterocarpus santalinus 2
Pterocarpus santalinus 6
Pterocarpus santalinus 8
Pterocarpus santalinus 10
Pterocarpus angolensis 1
Pterocarpus indicus 4
Pterocarpus macrocarpus 2
Pterocarpus indicus 5
Pterocarpus indicus 6
Pterocarpus indicus 1
Pterocarpus angolensis 6
Pterocarpus macrocarpus 5
63
53
79
51
55
50
54
99
68
0.005

h) ndhF-rpl32+ITS2

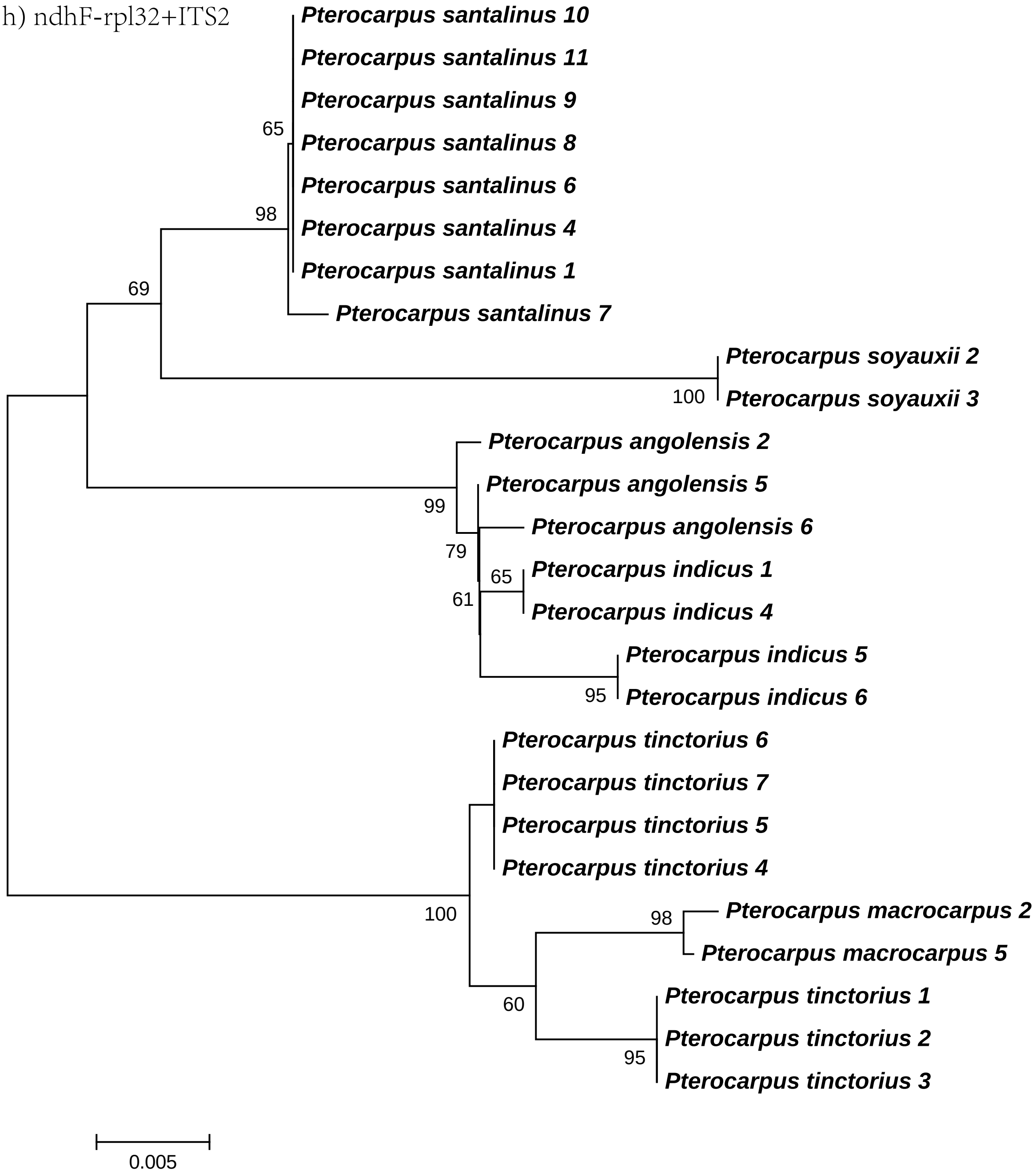

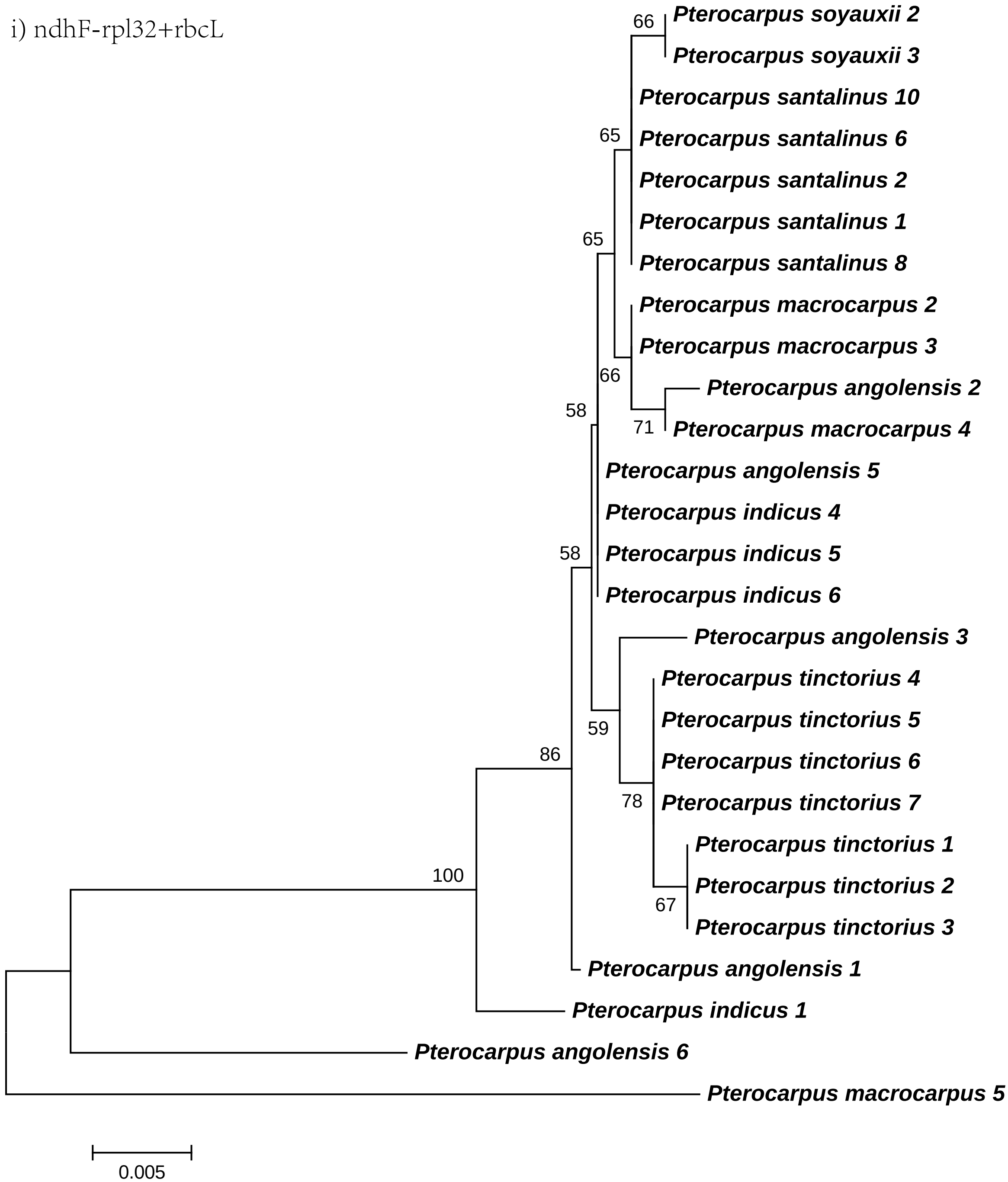
i) ndhF-rpl32+rbcL
Pterocarpus soyauxii 2
Pterocarpus soyauxii 3
Pterocarpus santalinus 10
Pterocarpus santalinus 6
Pterocarpus santalinus 2
Pterocarpus santalinus 1
Pterocarpus santalinus 8
Pterocarpus macrocarpus 2
Pterocarpus macrocarpus 3
Pterocarpus angolensis 2
Pterocarpus macrocarpus 4
Pterocarpus angolensis 5
Pterocarpus indicus 4
Pterocarpus indicus 5
Pterocarpus indicus 6
Pterocarpus angolensis 3
Pterocarpus tinctorius 4
Pterocarpus tinctorius 5
Pterocarpus tinctorius 6
Pterocarpus tinctorius 7
Pterocarpus tinctorius 1
Pterocarpus tinctorius 2
Pterocarpus tinctorius 3
Pterocarpus angolensis 1
Pterocarpus indicus 1
Pterocarpus angolensis 6
Pterocarpus macrocarpus 5
66
65
65
66
71
58
58
59
86
78
67
100
0.005

j) rbcL+ITS2

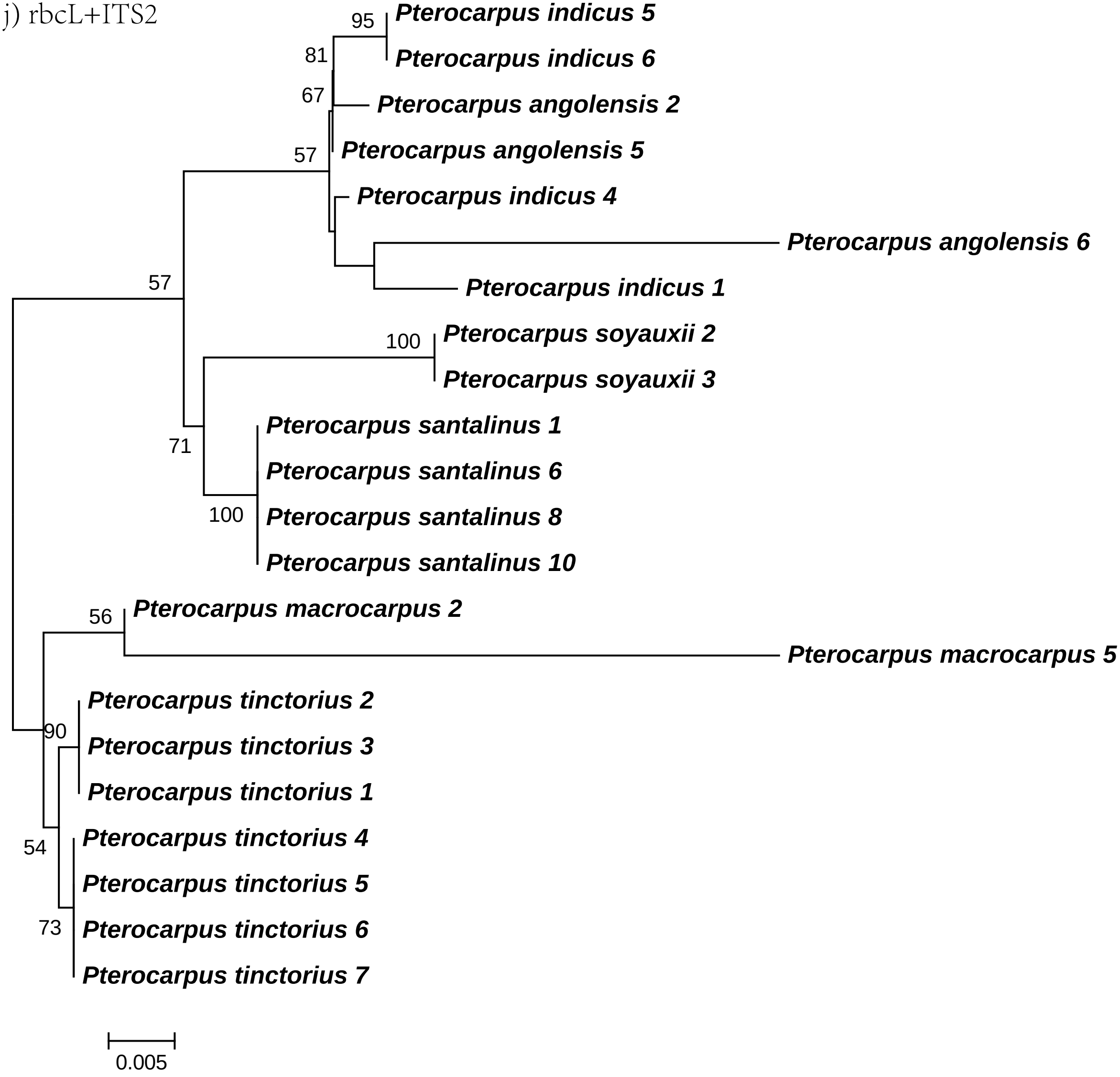

Pterocarpus indicus 5
Pterocarpus indicus 6
Pterocarpus angolensis 2
Pterocarpus angolensis 5
Pterocarpus indicus 4
Pterocarpus angolensis 6
Pterocarpus indicus 1
Pterocarpus soyauxii 2
Pterocarpus soyauxii 3
Pterocarpus santalinus 1
Pterocarpus santalinus 6
Pterocarpus santalinus 8
Pterocarpus santalinus 10
Pterocarpus macrocarpus 2
Pterocarpus macrocarpus 5
Pterocarpus tinctorius 2
Pterocarpus tinctorius 3
Pterocarpus tinctorius 1
Pterocarpus tinctorius 4
Pterocarpus tinctorius 5
Pterocarpus tinctorius 6
Pterocarpus tinctorius 7
95
81
67
57
57
100
71
100
56
90
54
73
0.005

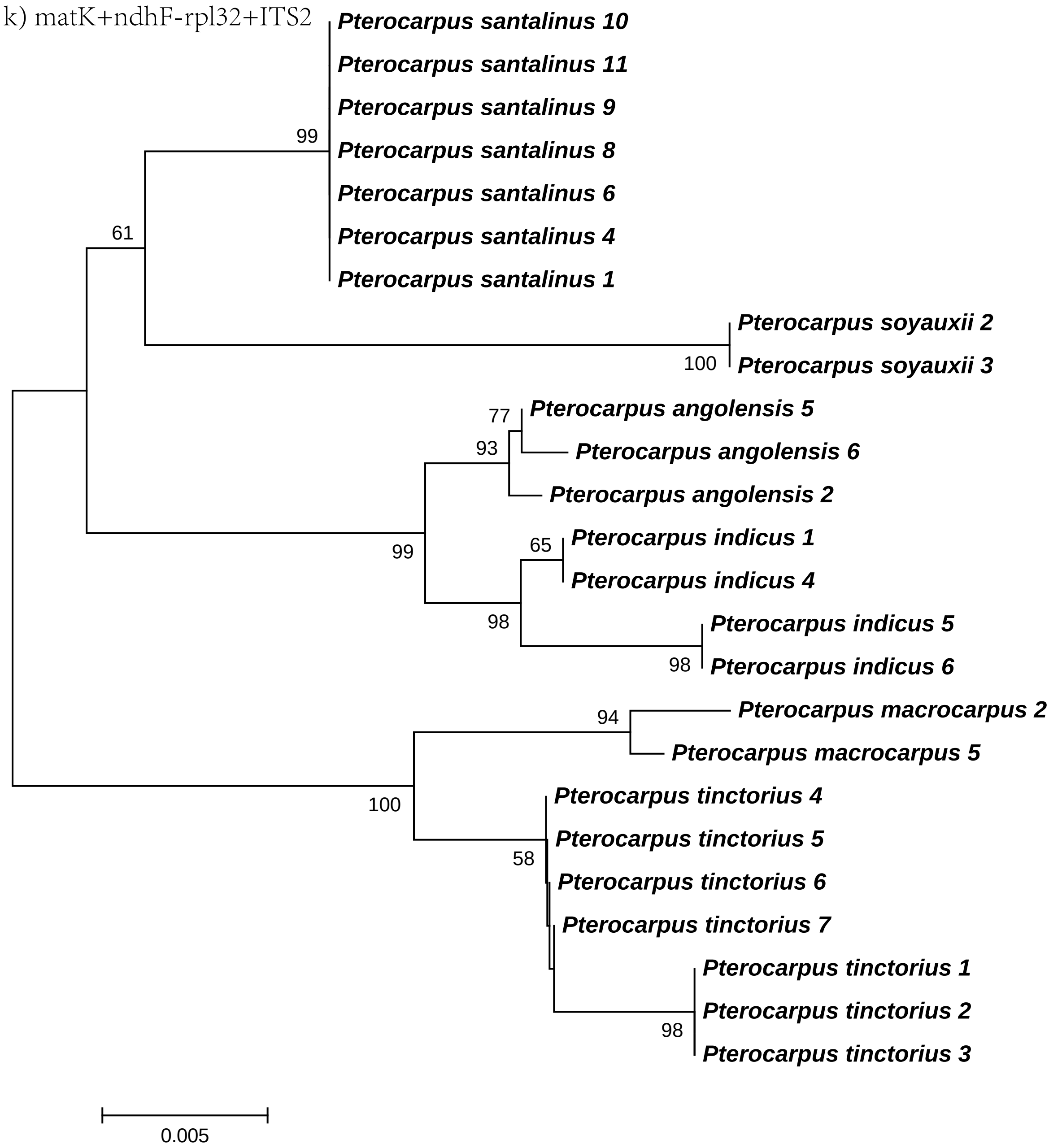
k) matK+ndhF-rpl32+ITS2
Pterocarpus santalinus 10
Pterocarpus santalinus 11
Pterocarpus santalinus 9
Pterocarpus santalinus 8
Pterocarpus santalinus 6
Pterocarpus santalinus 4
Pterocarpus santalinus 1
Pterocarpus soyauxii 2
Pterocarpus soyauxii 3
Pterocarpus angolensis 5
Pterocarpus angolensis 6
Pterocarpus angolensis 2
Pterocarpus indicus 1
Pterocarpus indicus 4
Pterocarpus indicus 5
Pterocarpus indicus 6
Pterocarpus macrocarpus 2
Pterocarpus macrocarpus 5
Pterocarpus tinctorius 4
Pterocarpus tinctorius 5
Pterocarpus tinctorius 6
Pterocarpus tinctorius 7
Pterocarpus tinctorius 1
Pterocarpus tinctorius 2
Pterocarpus tinctorius 3
99
61
100
77
93
99
65
98
98
94
100
58
98
0.005

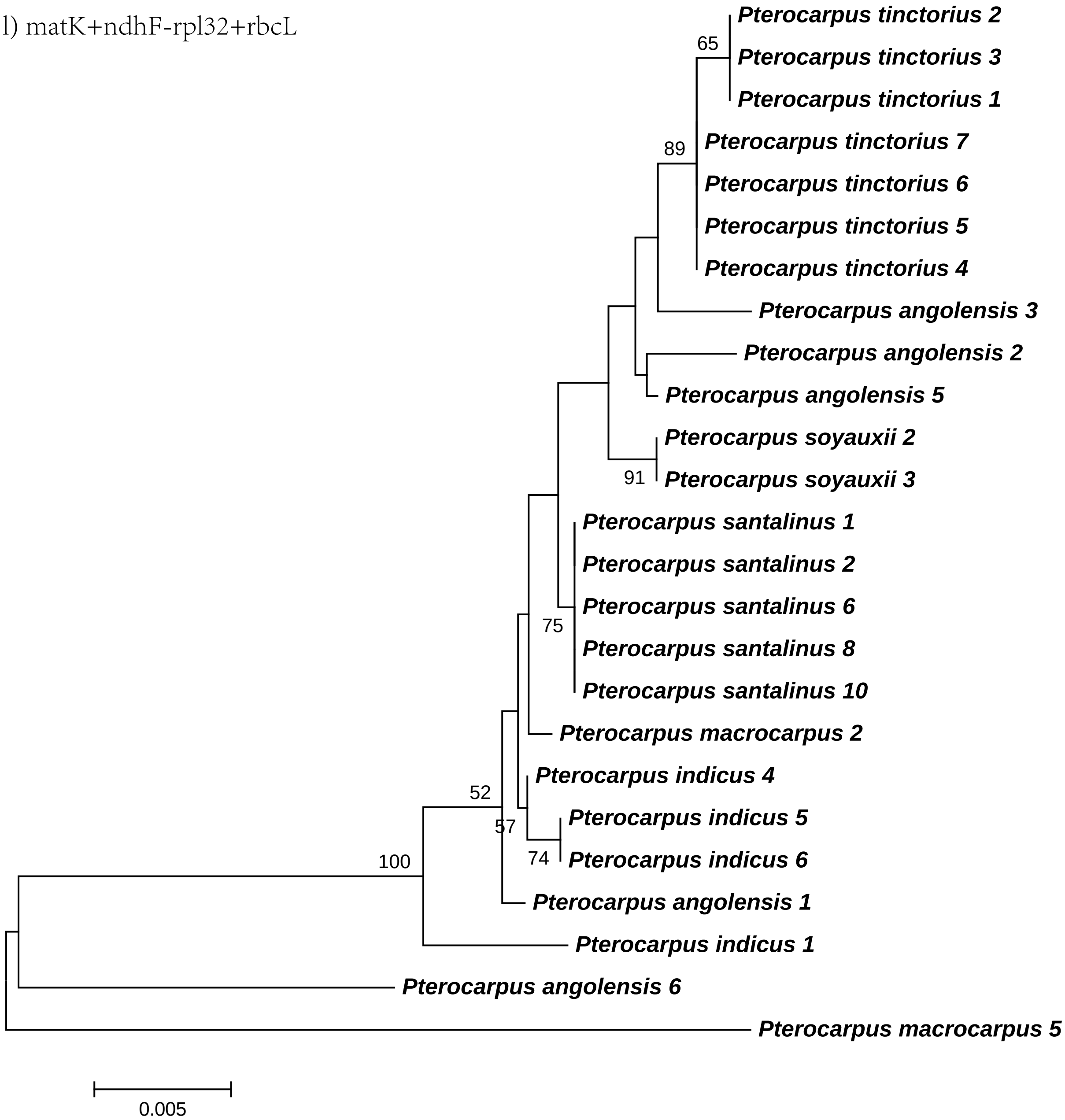

l) matK+ndhF-rpl32+rbcL
Pterocarpus tinctorius 2
Pterocarpus tinctorius 3
Pterocarpus tinctorius 1
Pterocarpus tinctorius 7
Pterocarpus tinctorius 6
Pterocarpus tinctorius 5
Pterocarpus tinctorius 4
Pterocarpus angolensis 3
Pterocarpus angolensis 2
Pterocarpus angolensis 5
Pterocarpus soyauxii 2
Pterocarpus soyauxii 3
Pterocarpus santalinus 1
Pterocarpus santalinus 2
Pterocarpus santalinus 6
Pterocarpus santalinus 8
Pterocarpus santalinus 10
Pterocarpus macrocarpus 2
Pterocarpus indicus 4
Pterocarpus indicus 5
Pterocarpus indicus 6
Pterocarpus angolensis 1
Pterocarpus indicus 1
Pterocarpus angolensis 6
Pterocarpus macrocarpus 5
65
89
91
75
52
57
74
100
0.005

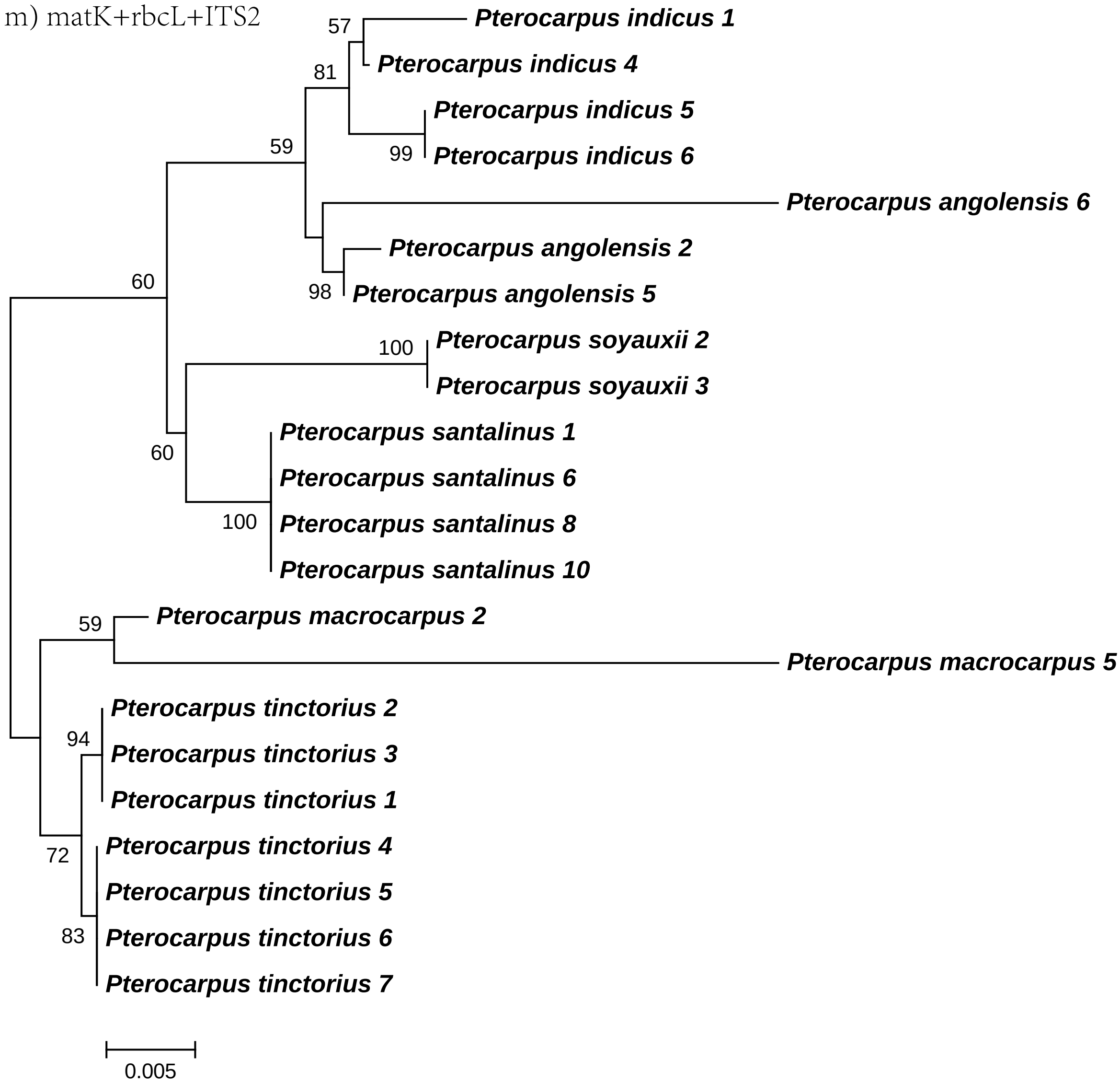
m) matK+rbcL+ITS2
57
Pterocarpus indicus 1
Pterocarpus indicus 4
81
Pterocarpus indicus 5
59
99
Pterocarpus indicus 6
Pterocarpus angolensis 6
Pterocarpus angolensis 2
60
98
Pterocarpus angolensis 5
100
Pterocarpus soyauxii 2
Pterocarpus soyauxii 3
Pterocarpus santalinus 1
60
Pterocarpus santalinus 6
100
Pterocarpus santalinus 8
Pterocarpus santalinus 10
59
Pterocarpus macrocarpus 2
Pterocarpus macrocarpus 5
Pterocarpus tinctorius 2
94
Pterocarpus tinctorius 3
Pterocarpus tinctorius 1
72
Pterocarpus tinctorius 4
Pterocarpus tinctorius 5
83
Pterocarpus tinctorius 6
Pterocarpus tinctorius 7
0.005

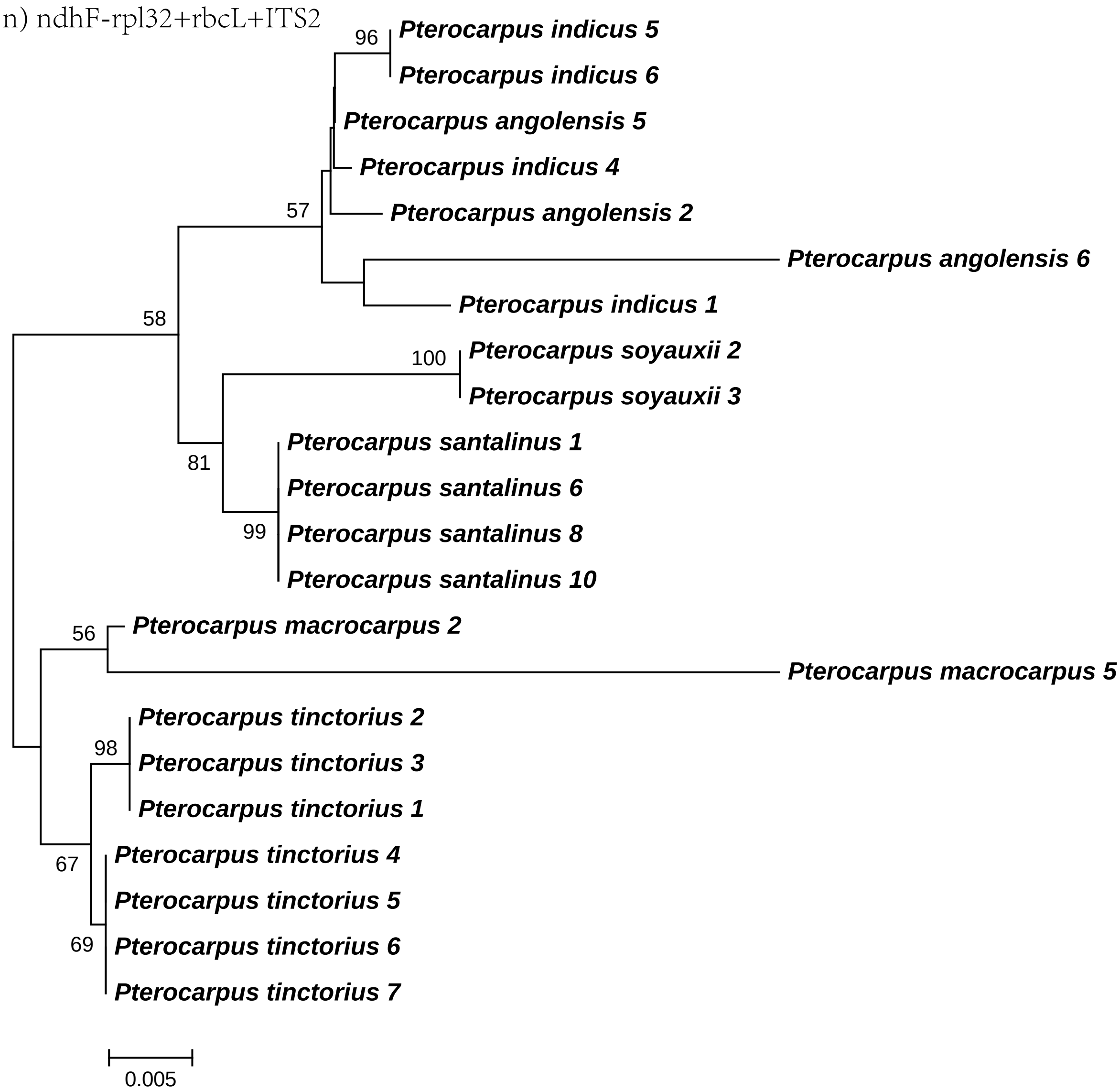
n) ndhF-rpl32+rbcL+ITS2
96
Pterocarpus indicus 5
Pterocarpus indicus 6
Pterocarpus angolensis 5
Pterocarpus indicus 4
57
Pterocarpus angolensis 2
Pterocarpus angolensis 6
Pterocarpus indicus 1
58
100
Pterocarpus soyauxii 2
Pterocarpus soyauxii 3
81
Pterocarpus santalinus 1
Pterocarpus santalinus 6
99
Pterocarpus santalinus 8
Pterocarpus santalinus 10
56
Pterocarpus macrocarpus 2
Pterocarpus macrocarpus 5
98
Pterocarpus tinctorius 2
Pterocarpus tinctorius 3
Pterocarpus tinctorius 1
67
Pterocarpus tinctorius 4
Pterocarpus tinctorius 5
69
Pterocarpus tinctorius 6
Pterocarpus tinctorius 7
0.005

o) matK+ndhF-rpl32+rbcL+ITS2

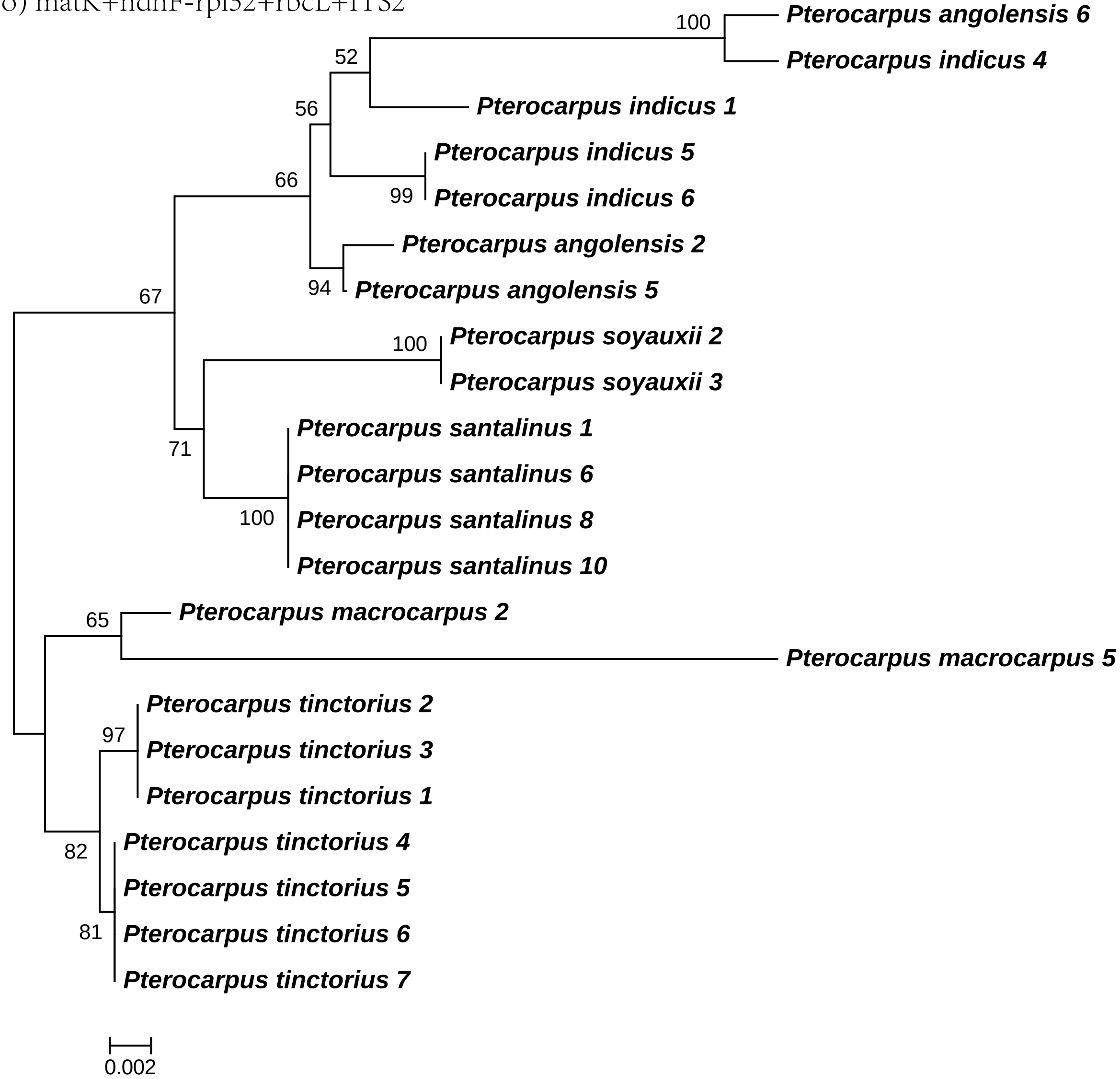

Supplement: Supplementary file 1 — Supplementary information [file 41598_2018_20381_MOESM1_ESM.pdf]
